# Exposure-aware multimodal engagement prediction for short-video platforms: an explainable human–media interaction framework

**DOI:** 10.3389/frai.2026.1896199

**Published:** 2026-08-25

**Authors:** Jiahao Zhang, Huan Liu

**Affiliations:** The Education University of Hong Kong, Hong Kong, Hong Kong SAR, China

**Keywords:** algorithmic amplification, explainable artificial intelligence, exposure-aware modeling, human–media interaction, multimodal fusion, recommender systems, SHAP, short-video platforms

## Abstract

Short-video recommendation feeds are increasingly important human–media interaction systems. However, the observable pathways through which algorithmic exposure is associated with multimodal content and measurable user engagement remain insufficiently explained. Existing recommender-system studies usually optimize ranking accuracy or watch-time objectives. Communication-oriented studies often examine engagement without modeling the exposure conditions under which interaction data are generated. This paper develops an explainable exposure-aware multimodal framework for short-video engagement prediction and observable algorithmic amplification measurement. The framework combines the Algorithmic Amplification Gap (AAG), random-exposure baselines, multi-target engagement modeling, exposure-gated multimodal fusion, and interpretable feature attribution. KuaiRand-Pure is used as the main exposure-aware dataset, containing 27,285 users, 7,551 short videos, and 1,436,609 user–video interaction records. KuaiRec is used as an auxiliary robustness benchmark. MicroLens is introduced as an external raw-multimodal validation dataset with titles, cover images, audio, and full-length micro-videos. Across experiments, exposure proxies and temporally separated early-window feedback signals provide substantial explanatory power. Textual features, semantic/category proxy features, temporal/retention-behavioral proxy features, and raw multimodal representations remain important for target-specific engagement patterns. The proposed Exposure-Gated Multimodal Fusion component achieves statistically reliable but incremental gains over concatenation-based fusion. The main contribution is therefore an auditable human–media interaction protocol for examining how observable algorithmic visibility, multimodal content, and user feedback jointly shape short-video engagement.

## 1 Introduction

Short-video recommendation feeds have become a large-scale human–media interaction interface. Users do not only search for information or follow pre-selected channels in this interface. They continuously interact with algorithmically ranked videos whose visibility is updated according to content representations, contextual signals, and early behavioral feedback. This setting creates a difficult computer-science problem. Engagement is not generated by content alone, and it is not fully explained by a proprietary ranking function. Rather, it emerges from a coupled content–exposure–feedback process in which multimodal content cues, platform-visible metadata, exposure intensity, and user reactions reinforce or suppress one another over time.

For this submission to Frontiers in Computer Science, the problem is relevant to human–media interaction, recommender systems, explainable artificial intelligence, and multimodal learning. Existing recommender-system studies have made major progress in ranking accuracy, watch-time prediction, and exposure-bias reduction. Multimedia-computing studies have also shown that visual, textual, acoustic, and temporal cues can improve short-video performance prediction. However, many models still treat engagement prediction mainly as an optimization task. Less attention has been paid to whether the same modeling pipeline can explain how observable exposure conditions change the measured relationship between content features and user behavior. This gap is particularly important for short-video platforms because small early interaction differences may be associated with large visibility differences through repeated recommendation.

This study develops an explainable exposure-aware multimodal framework for short-video engagement prediction and observable algorithmic amplification measurement. The goal is not to reverse-engineer the internal ranking function of a commercial platform or to claim a large new recommender architecture. Instead, the paper proposes a reproducible human–media interaction protocol that combines random-exposure baselines, multi-target engagement modeling, exposure-conditioned multimodal fusion, and interpretable feature attribution. The framework explicitly separates content-side features from exposure-side variables so that the contribution of algorithmic visibility can be examined rather than hidden inside a black-box prediction score.

The empirical analysis uses three open-source short-video recommendation resources. KuaiRand-Pure is used as the main exposure-aware dataset because it contains both standard recommendation logs and random-exposure logs. KuaiRec is used as an auxiliary robustness benchmark under a different user–video interaction matrix. MicroLens is used as an external raw-multimodal validation dataset because it provides titles, cover images, audio, and full-length micro-videos. Together, these datasets make it possible to study both exposure-aware interaction logs and raw multimodal content representations.

The research is guided by four questions. RQ1 asks how textual cues, semantic/category proxy features, temporal/retention-behavioral proxy features, metadata, and raw multimodal representations affect short-video engagement outcomes. RQ2 asks whether exposure intensity and temporally separated early feedback provide additional explanatory power beyond content features alone. RQ3 asks whether an exposure-gated fusion mechanism improves prediction and interpretation compared with standard concatenation and tree-based baselines. RQ4 asks how algorithmic recommendation reshapes human–media interaction by turning audience feedback into a signal for subsequent content visibility.

The paper makes four contributions. First, it introduces the Algorithmic Amplification Gap (AAG), which estimates observable recommendation-associated amplification by comparing engagement observed under standard recommendation with a content-side baseline estimated from random-exposure logs. Second, it proposes an Exposure-Gated Multimodal Fusion (EGMF) component that uses visibility/context representations to regulate the contribution of textual, semantic, temporal/behavioral, and metadata feature groups. Third, it evaluates multiple engagement targets, including like rate, comment rate, share rate, favorite rate, and completion rate, instead of collapsing user behavior into a single popularity score. Fourth, it combines predictive modeling with SHAP attribution, modality ablation, partial-dependence analysis, and robustness checks, thereby making the framework useful for both model evaluation and mechanism-oriented explanation.

**Figure 1:**
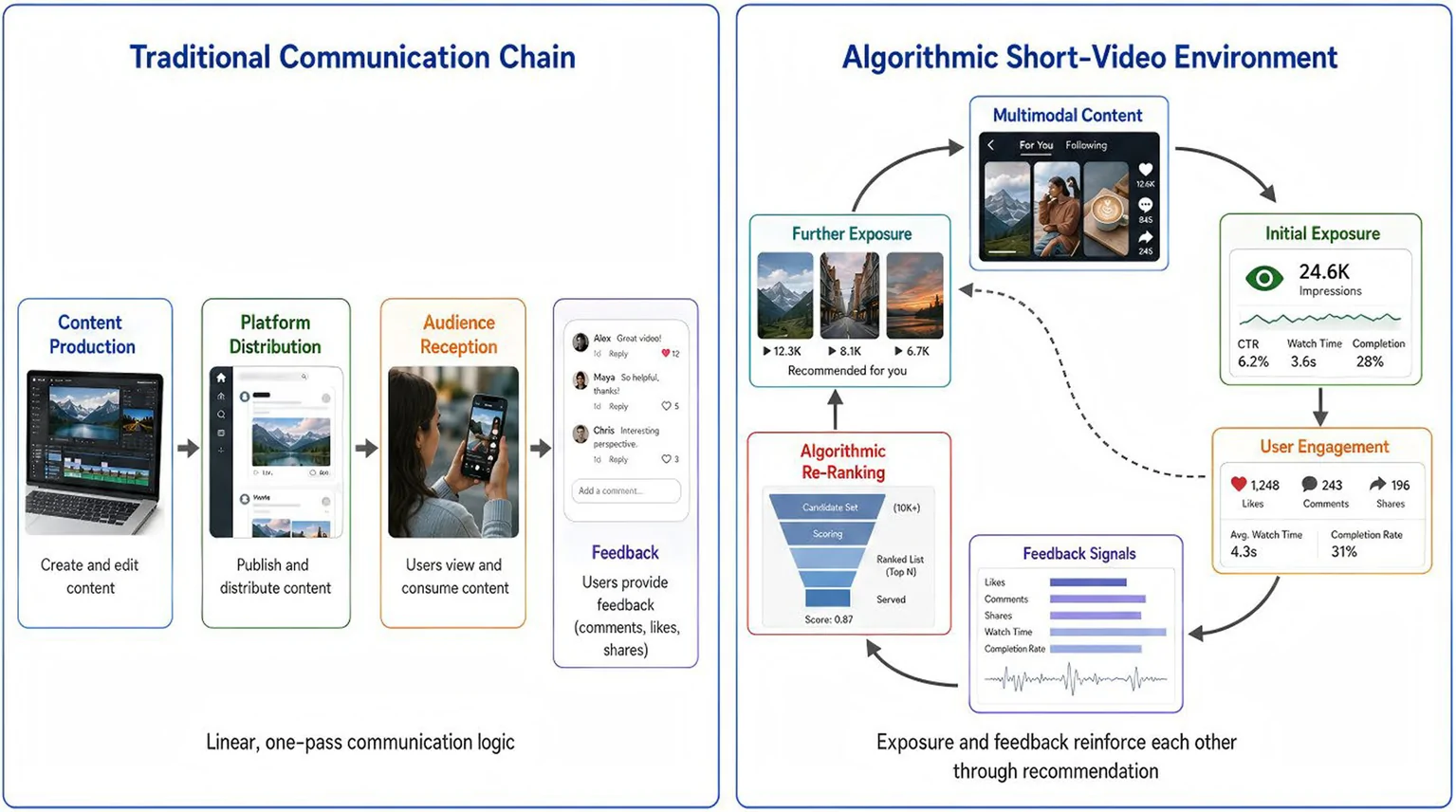
Motivation of the proposed study. This figure contrasts a conventional one-pass media distribution chain with the algorithmic short-video environment, in which multimodal content, exposure allocation, user feedback, and iterative re-ranking jointly form a recursive content–exposure–feedback loop. The comparison clarifies why short-video engagement should be modeled as a human–media interaction process rather than as a direct content-response relationship.

Figure 1 summarizes the motivation. In a conventional one-pass content distribution process, content is produced, distributed, and then evaluated by audience response. In algorithmic short-video environments, audience response becomes an input for subsequent recommendation, forming a recursive content–exposure–feedback loop. This loop motivates the exposure-aware modeling design used in this paper.

**Figure 2:**
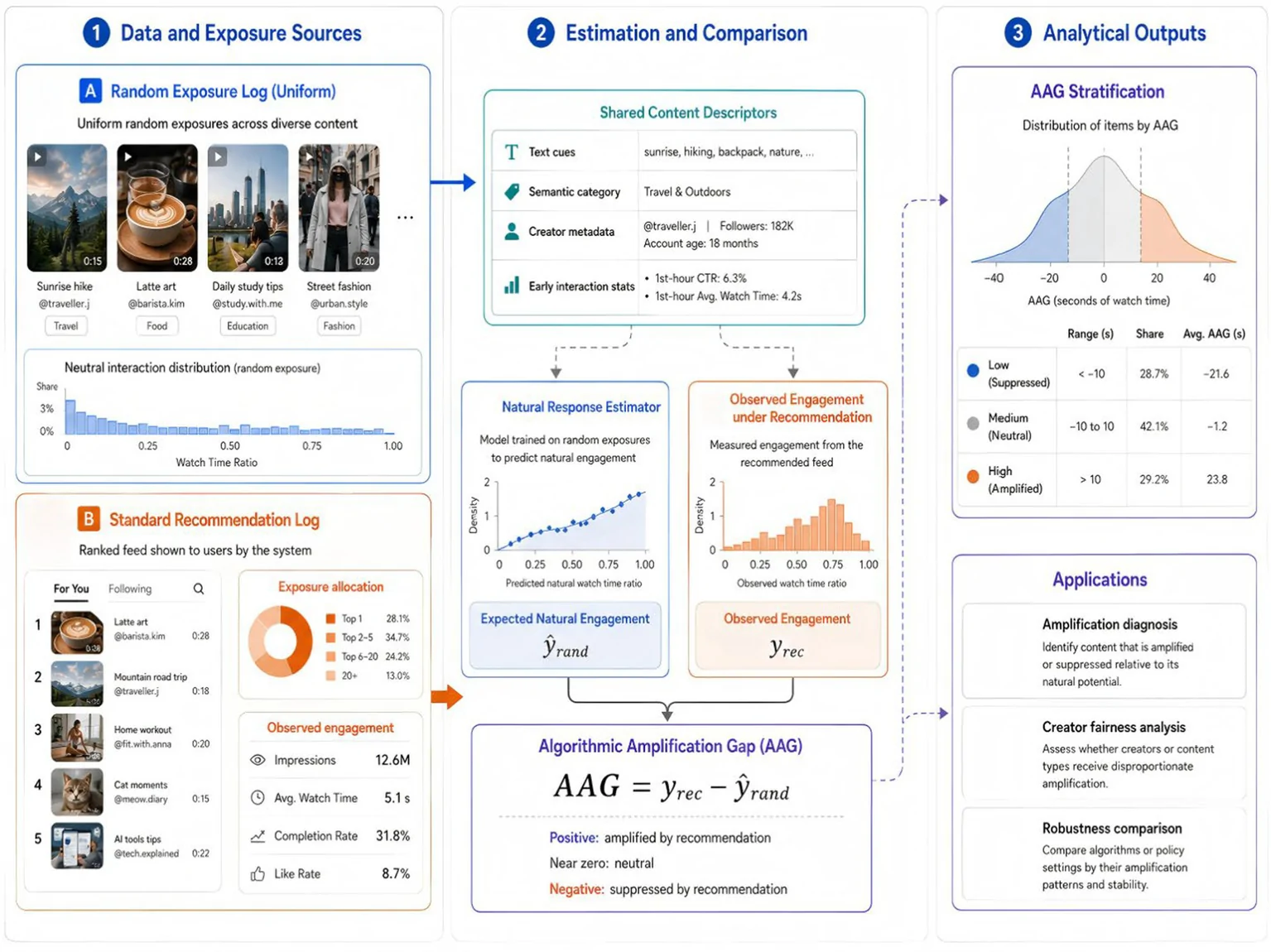
Mechanism design of the algorithmic amplification gap. The random-exposure branch estimates the natural engagement expected from content-side features, whereas the standard-recommendation branch records observed engagement under platform-mediated exposure. Their difference provides a reproducible proxy for observable recommendation-associated amplification.

Figure 2 summarizes the mechanism design of AAG. Instead of inferring algorithmic amplification only from standard recommendation logs, the proposed design uses random-exposure logs to estimate a content-side engagement baseline and then compares it with engagement observed under standard recommendation. The resulting gap provides a reproducible proxy for amplification without requiring access to proprietary ranking parameters.

## 2 Related work and human–media interaction framework

The proposed work is located at the intersection of human–media interaction, multimedia recommendation, and explainable machine learning. From a computer-science perspective, short-video platforms are not only distribution channels; they are adaptive interaction systems in which users, content items, and ranking mechanisms continuously exchange signals. Human–media interaction research provides the behavioral interpretation of user engagement, recommender-system research provides methods for modeling exposure allocation and feedback loops, and multimodal learning provides tools for representing heterogeneous video-side information.

This section reviews the research streams most relevant to the proposed framework: multimodal micro-video recommendation, exposure-bias datasets, watch-time debiasing, short-video engagement prediction, and computational studies of algorithmic mediation. The central limitation identified across these streams is that prediction accuracy, exposure correction, and human–media interpretation are often studied separately. The present paper connects them through an auditable protocol that treats exposure as a measurable interaction condition rather than as a nuisance variable.

Early multimedia recommendation studies demonstrate that micro-video recommendation cannot rely only on item identifiers or collaborative signals. MMGCN, for example, explicitly models modality-specific user preferences by using multi-modal graph convolution over user–item interactions (Wei et al., 2019). This insight is important for the present study because it implies that different users and videos may interact through different modalities: one video may be driven by semantic/category novelty, another by temporal rhythm, raw audio in MicroLens, or emotional title, and another by creator reputation. More recent surveys also show that multimodal recommender systems increasingly focus on modality fusion, representation alignment, missing-modality robustness, and cross-modal interaction (Liu et al., 2024). In short-video contexts, adaptive fusion is especially relevant because textual, visual, and audio signals are highly unequal across videos (Hu et al., 2023).

Another important literature concerns exposure bias and watch-time bias. Recommender systems are trained on logged interactions, but logged interactions are shaped by what the platform has already exposed. Therefore, observed engagement is not a pure measurement of user preference. KuaiRec provides a fully observed user–video interaction matrix for evaluating recommender systems under reduced missingness (Gao et al., 2022a), whereas KuaiRand introduces randomly exposed videos to reduce exposure bias in sequential recommendation (Gao et al., 2022b). Watch-time prediction further suffers from duration bias because long videos may receive different exposure and naturally produce different viewing durations. D2Q formalizes duration as a confounding factor and proposes a duration-deconfounded quantile framework for watch-time prediction (Zhan et al., 2022). These studies support our decision to model completion and watch-related metrics as normalized outcomes and to include duration and exposure proxies as control variables.

Short-video engagement prediction has also become an emerging topic in computer vision and multimedia quality assessment. Work on SnapUGC explores engagement prediction for newly published short videos and emphasizes the role of visual content, background music, and textual metadata (Li et al., 2024). Large multimodal models have recently been used to predict short-video engagement by jointly processing video frames, text metadata, and background sound (Sun et al., 2025). These studies demonstrate the technical feasibility of multimodal prediction, but they are less focused on the human–media interaction interpretation of algorithmic amplification. In contrast, our study treats prediction as a means to explain how content cues and exposure proxies jointly shape user participation.

MicroLens is especially relevant to the present revision because it provides a public short-video recommendation benchmark with raw modality information. Unlike KuaiRand-Pure and KuaiRec, which are strongest for exposure-aware recommendation logs but do not release original video frames or audio waveforms, MicroLens includes titles, cover images, audio, and full-length micro-videos (Ni et al., 2023). Therefore, this study uses KuaiRand-Pure for the main AAG and exposure-mediation analysis, while MicroLens is used as an external raw-multimodal validation dataset to test whether EGMF also benefits from original visual and acoustic representations.

Human-computer interaction research on TikTok also highlights the importance of real-world engagement data. A recent CHI study analyzed user engagement with TikTok’s short-format video recommendations through data donations, showing that recommendation feeds can be studied empirically even when platform algorithms remain opaque (Zannettou et al., 2024). This provides a useful methodological lesson: because internal ranking models are rarely accessible, researchers can use observable recommendation outputs and behavioral traces to infer algorithmic mediation cautiously. Accordingly, the present study does not claim to identify the proprietary platform algorithm. Instead, it operationalizes algorithmic exposure through observable proxies such as exposure intensity, early interaction rate, repeated exposure, recommendation source, and time to first engagement, while content similarity is retained as a separate content-matching covariate.

Recent entity-centric news recommendation studies also provide useful methodological inspiration for this framework, even though the present task concerns short-video rather than news recommendation. SentiEntityRec incorporates entity-level sentiment vectors into graph-based news representations, showing how fine-grained affect can be attached to semantic entities (Wang et al., 2025). KnowEntityRec uses an entity-centric knowledge perception module to augment recommendation with dynamic knowledge-graph signals (Wang et al., 2026a). HPNewsRec further combines semantic, sentiment-aware, and knowledge-graph information in a hybrid perception architecture (Wang et al., 2026b). These studies support the broader idea that engagement prediction can benefit from entity-aware semantics, sentiment perception, and hybrid information fusion. In the current short-video setting, these ideas motivate future extensions that would enrich captions, cover text, creator metadata, and category labels with entity-level sentiment and knowledge-aware representations when such annotations are available.

Table 1 compares representative research streams and positions the current study. The main gap is that many studies emphasize either recommendation accuracy or communication interpretation, while fewer studies integrate multimodal content, exposure proxies, multi-target engagement prediction, and explainable machine learning into one framework.

**Table 1:** Positioning of the proposed framework relative to representative research streams.

Research stream	Typical focus	Main variables	Strength
Multimodal micro-video recommendation	Ranking and personalization	User–item interactions, textual, semantic/category, and temporal/retention-behavioral proxy features	Models modality-specific preference and improves recommendation accuracy
Exposure-bias datasets	Evaluation under biased or randomized exposure	Logged impressions, clicks, watch time, feedback signals	Highlights that observed engagement depends on exposure allocation
Watch-time debiasing	Duration and watch-time prediction	Video duration, watch time, completion, duration buckets	Controls duration bias in video recommendation
Short-video engagement prediction	Cold-start and UGC performance prediction	Visual quality, music, metadata, captions	Captures rich multimodal signals
Computational communication	Platform governance and algorithmic mediation	Topics, sentiments, interactions, visibility	Interprets platform influence on public communication
This study	Explainable engagement mechanism	Text, semantic/category proxy features, temporal/retention-behavioral proxy features, metadata, exposure proxies, multi-target engagement	Connects multimodal content, recommendation exposure, and user feedback

The table synthesizes the main analytical traditions relevant to this study and highlights how the proposed framework differs from prior work by jointly modeling multimodal content, algorithmic exposure, and multi-dimensional engagement in a single explainable pipeline.

The differentiation from prior work is threefold. First, the paper is not positioned as another end-to-end recommender architecture; rather, it provides a reproducible, mechanism-oriented measurement framework for explaining engagement under observable recommendation exposure. Existing multimodal recommender studies usually optimize ranking accuracy, whereas the present study separates content-side proxies, exposure-side proxies, and feedback outcomes so that the role of algorithmic visibility can be examined explicitly. Second, the framework uses open benchmark data and transparent exposure-proxy equations instead of an undocumented platform crawl, which improves reproducibility for communication and media analytics research. Third, the study reports target-specific explanations across like, comment, share, favorite, and completion behaviors, showing that different forms of engagement are driven by different combinations of content semantics, retention proxies, metadata, and exposure conditions. Thus, the contribution is methodological and explanatory rather than purely architectural: it transforms short-video recommendation benchmarks into an auditable protocol for human–media interaction analysis. The EGMF component is deliberately lightweight so that any observed gain can be interpreted in relation to exposure-conditioned feature weighting rather than attributed to an opaque large model.

**Figure 3:**
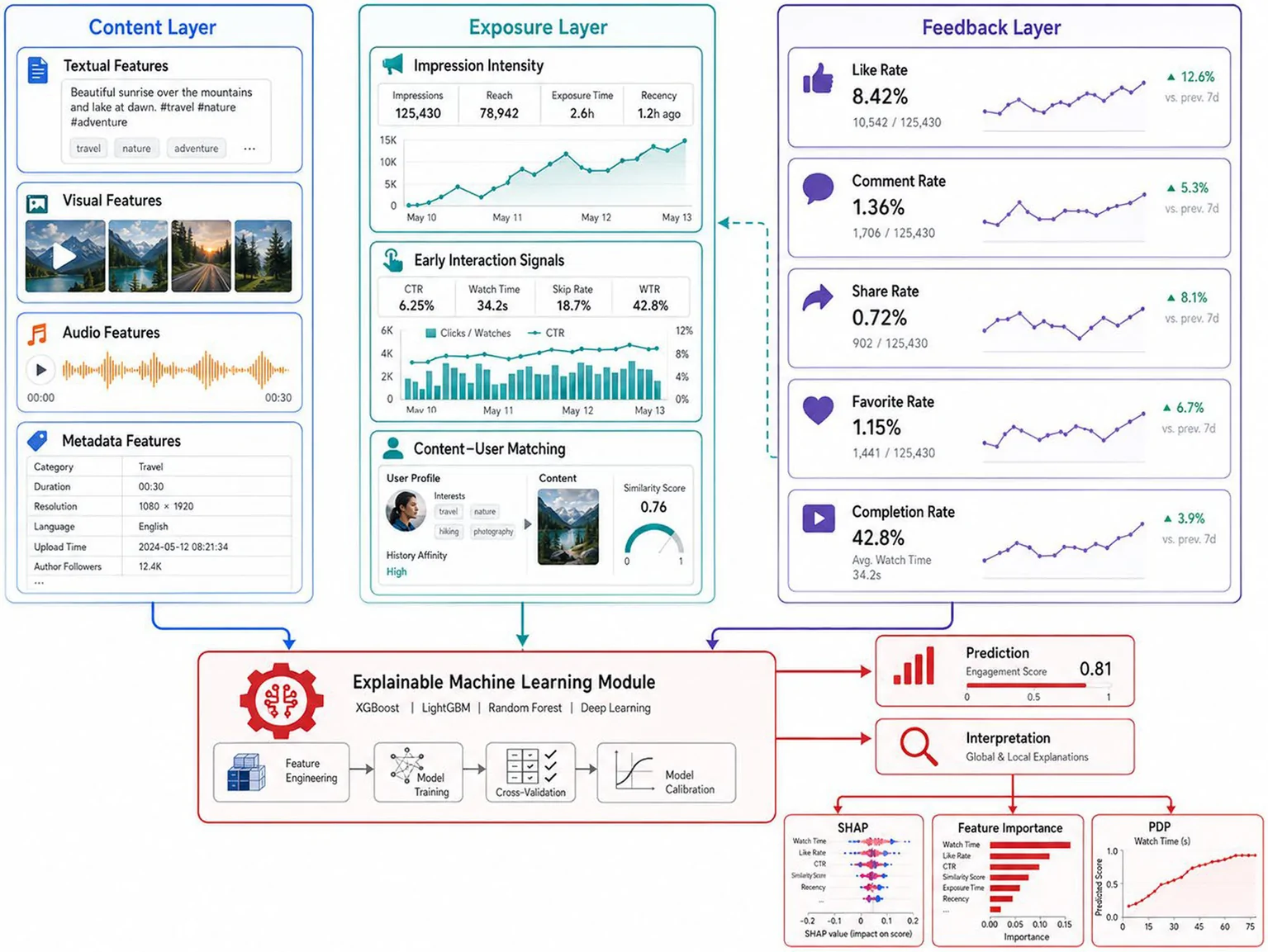
Exposure-gated multimodal framework for explainable short-video engagement modeling. The figure integrates multimodal content signals, algorithmic exposure proxies, multiple engagement targets, and the explainable modeling module used in the empirical analysis. It visualizes the core analytical logic of the paper, showing how heterogeneous inputs are transformed into a unified representation for prediction, comparison, and interpretation.

The proposed research framework is shown in Figure 3. It consists of three conceptual layers. The first layer is multimodal content representation, which includes textual, semantic/category proxy, temporal/retention-behavioral proxy, and metadata variables. The second layer is algorithmic exposure, which includes observable proxies of how a video enters and circulates in a recommendation feed. The third layer is user engagement, measured as multiple normalized outcomes. The framework assumes that content features influence engagement both directly and indirectly through exposure allocation. This assumption is consistent with the logic of algorithmic amplification: videos that generate stronger initial response may receive more subsequent exposure, and subsequent exposure may enlarge differences in engagement.

Based on this framework, the paper formulates four hypotheses. H1: multimodal content features significantly predict user engagement, but different modalities contribute differently to different engagement targets. H2: exposure proxies and early feedback signals provide incremental explanatory power beyond content features alone. H3: the interaction between content attractiveness and exposure intensity is associated with an observable amplification pattern, such that high-quality or emotionally salient content obtains stronger engagement under higher exposure conditions. H4: explainable machine-learning models can reveal modality-specific patterns that correspond to communication concepts such as attention, affective appeal, social conversation, and platform visibility.

## 3 Materials and methods

**Figure 4:**
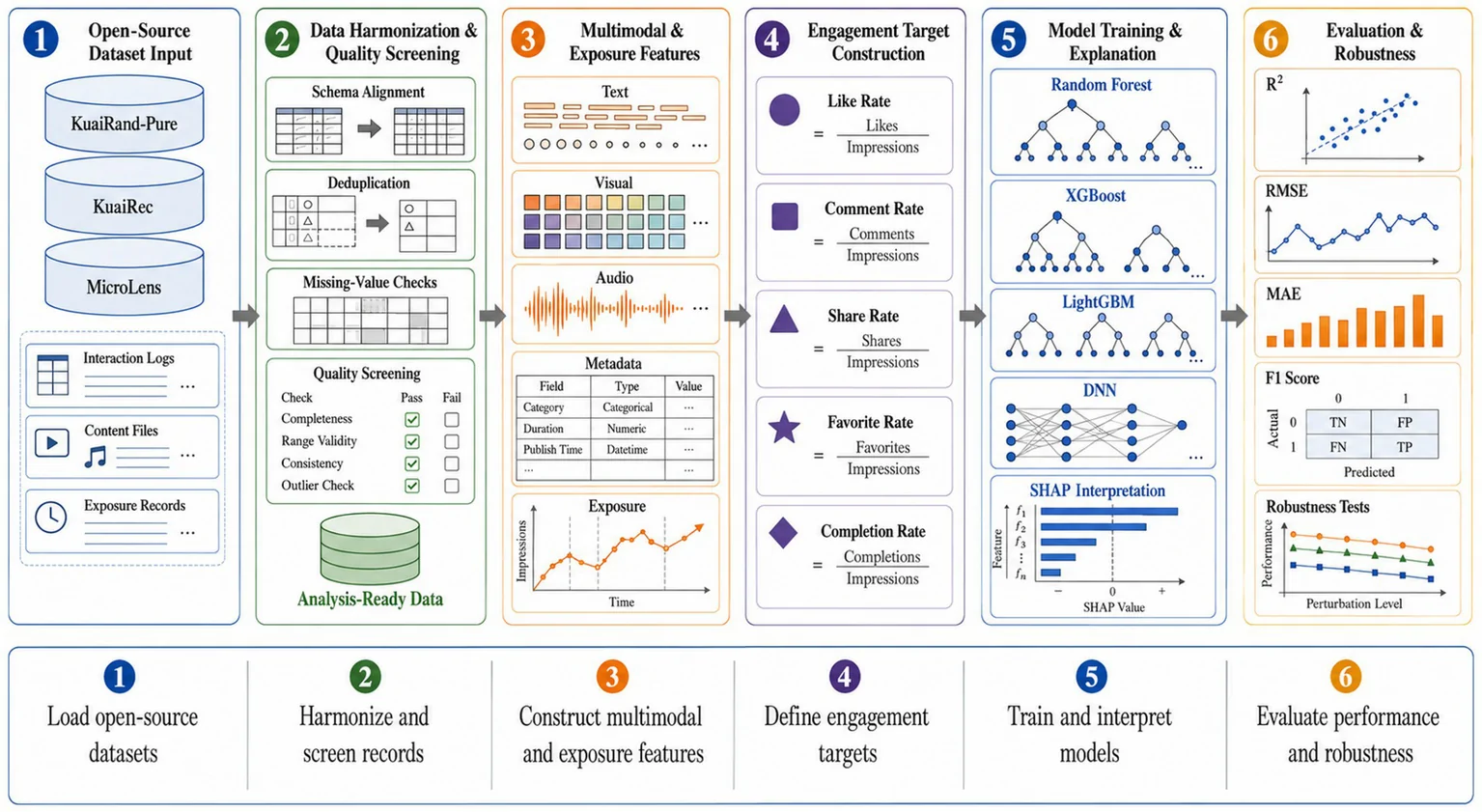
Methodological pipeline from public short-video sampling to explainable engagement modeling. The workflow summarizes the end-to-end research procedure, including data acquisition, multimodal preprocessing, feature engineering, target construction, model training, evaluation, and *post hoc* interpretation. It also clarifies how empirical execution is linked to the theoretical objectives of the study.

The proposed methodology is designed as a reproducible human–media interaction and machine-learning pipeline. It starts with public short-video records, extracts five groups of features, constructs engagement targets, trains multiple predictive models, and then interprets the models through feature attribution and robustness tests. The workflow is shown in Figure 4.

### 3.1 Data design and variable construction

The empirical design is based on open-source short-video recommendation datasets rather than an unspecified platform crawl. Three datasets are used with different roles. KuaiRand-Pure is the main exposure-aware dataset because it contains both standard recommendation logs and random-exposure intervention logs, which are required to construct the Algorithmic Amplification Gap (AAG). KuaiRec is used as an auxiliary recommendation benchmark for robustness checks under a different user–video interaction matrix. MicroLens is introduced as an external raw-multimodal validation dataset because it provides original modality information, including titles, cover images, audio, and full-length micro-videos (Gao et al., 2022a, 2022b; Ni et al., 2023).

The final analytical dataset constructed from KuaiRand-Pure contains 27,285 users, 7,551 short videos, and 1,436,609 user–video interaction records. The observation window spans from 8 April 2022 to 8 May 2022. Specifically, the standard recommendation logs cover 8 April 2022 to 21 April 2022, while the standard recommendation and random-exposure logs cover 22 April 2022 to 8 May 2022. The dataset provides 30 user features, 62 item features, and 12 feedback signals, including click, like, comment, follow, forward/share, hate, play duration, completion-related behavior, and other user feedback indicators. The main files used in this study are log_standard_4_08_to_4_21_pure.csv, log_standard_4_22_to_5_08_pure.csv, log_random_4_22_to_5_08_pure.csv, user_features_pure.csv, video_features_basic_pure.csv, and video_features_statistic_pure.csv. The official supplementary files kuairand_video_captions.csv and kuairand_video_categories.csv are joined by the shared video identifier where available. These files provide captions, cover text, and category information and therefore allow the construction of textual and category-aware features without relying on undocumented web crawling.

To reduce dependence on a single recommendation benchmark, KuaiRec is additionally used as an auxiliary cross-dataset robustness benchmark. KuaiRec is also released from Kuaishou recommendation logs and is useful because it contains a nearly fully observed user–video interaction matrix. Its small matrix contains 1,411 users, 3,327 videos, and 4,676,570 interactions with 99.6% density, while its big matrix contains 7,176 users, 10,728 videos, and 12,530,806 interactions with 16.3% density. KuaiRec also includes user features, item categories, social-network information, and item-level daily statistics from 5 July 2020 to 5 September 2020. Because KuaiRec does not provide exactly the same multi-target feedback structure as KuaiRand-Pure, it is used mainly for robustness checks related to watch-ratio/completion modeling, item-category heterogeneity, and exposure-density sensitivity, rather than as the primary source of the multi-target engagement results.

MicroLens is used as an external raw-multimodal validation dataset. The full MicroLens benchmark contains 1 billion user–item interaction behaviors, 34 million users, and 1 million micro-videos, and it releases raw modality information including titles, cover images, audio, and full-length videos. For reproducible validation within a manageable experimental scale, this study uses the public MicroLens-100 K release, which provides interaction-pair files, title files, like/view statistics, comment files, cover images, sampled frame archives, extracted modality features, and download scripts for the corresponding videos. MicroLens is not used to construct AAG because it does not provide the same standard-versus-random exposure intervention structure as KuaiRand-Pure. Instead, it is used for a separate raw-multimodal validation of the fusion architecture. In this setting, the gate input is not the KuaiRand exposure representation. It is a benchmark-specific visibility/context representation constructed only from training-split item statistics and non-target context variables, such as historical item interaction volume, historical view count, item/category frequency, duration, and modality-availability indicators. Validation or test labels are not used to construct the gate. Because the public MicroLens validation files do not provide engagement labels fully equivalent to KuaiRand’s share and favorite variables, the MicroLens experiment reports the comparable targets available in that benchmark, namely like rate, comment rate, watch-ratio proxy, and a composite engagement score.

Table 2 summarizes the dataset configuration and reproducibility design. All datasets used in this study are open-source benchmarks, and no private user profiles, private messages, follower lists, or personally identifiable audience information are collected by the authors. User identifiers and video identifiers are anonymized or re-indexed in the released datasets. The unit of analysis in the main KuaiRand-Pure experiments is a video-level observation obtained by aggregating interaction-level records over the observation window, whereas the MicroLens validation uses item-level multimodal representations and interaction-derived engagement labels.

**Table 2:** Open-source datasets and reproducibility information.

Dataset	Scale and period	Available information	Role
KuaiRand-Pure	27,285 users, 7,551 videos, 1,436,609 interactions; 8 April 2022–8 May 2022	Standard recommendation logs, random-exposure logs, timestamps, user features, item features, video statistics, and 12 feedback signals	Main AAG and exposure-aware dataset
KuaiRand supplementary content files	Joined by video identifier to KuaiRand videos where available	Video captions, cover text, and video category labels	Textual and category enrichment for KuaiRand
KuaiRec small matrix	1,411 users, 3,327 videos, 4,676,570 interactions; 99.6% density	Watch ratio, play duration, video duration, item categories, user features, and social-network information	Fully observed robustness benchmark
KuaiRec big matrix	7,176 users, 10,728 videos, 12,530,806 interactions; 16.3% density	User–video interactions, item daily statistics, categories, and side information	Exposure-density sensitivity and cross-dataset validation
MicroLens-100 K/MicroLens	Full benchmark: 1 billion interactions, 34 million users, 1 million micro-videos; public 100 K release used for validation	Titles, comments, cover images, sampled video frames, audio extraction scripts, full-length videos, like/view statistics, and extracted multimodal features	External raw-multimodal validation

The table specifies the exact short-video recommendation datasets used in this study, the available fields, the observation periods or scale information, and the role of each dataset in the empirical design.

Because the datasets differ in modality availability, feature construction is dataset-specific. In KuaiRand-Pure and KuaiRec, the released files do not contain original video frames or audio waveforms. Therefore, the main exposure-aware experiments do not claim pixel-level visual extraction or waveform-level audio extraction. In these two datasets, visual- and audio-related information is represented conservatively as semantic/category proxy features and temporal/retention-behavioral proxy features. These include category labels, cover text, duration, play duration, completion behavior, watch ratio, retention-related statistics, and item-level aggregate statistics. In MicroLens, by contrast, raw video content is available. The external validation therefore extracts textual representations from titles and comments, visual representations from covers and sampled frames, and acoustic representations from extracted audio tracks or released modality features. These raw modality representations are passed through the same gated-fusion architecture, but the gate input is dataset-specific: KuaiRand-Pure uses exposure proxies derived from recommendation and random-exposure logs, whereas MicroLens uses a visibility/context gate derived from training-split item popularity, item/category frequency, duration, and modality-availability variables. This terminology prevents the KuaiRand-Pure and KuaiRec analyses from being read as direct computer-vision or audio-signal experiments, while still allowing MicroLens to test the raw-multimodal extension.

For video i , the primary engagement targets are constructed from the video-level statistics and aggregated interaction logs. Let $\mathrm{sho}w_{i}$ denote the number of impressions, $\mathrm{pla}y_{i}$ the number of plays, $\mathrm{lik}e_{i}$ the number of likes, $\text{commen}t_{i}$ the number of comments, $sh\mathrm{ar}e_{i}$ the number of forwards or shares, $\text{collec}t_{i}$ the number of collections or favorites, and $\text{complet}e_{i}$ the number of complete plays. To avoid leakage from early feedback variables into the target, each observation is split into an early window and a subsequent outcome window whenever timestamped interactions are available. Early-window counts are used only to construct feedback-loop predictors, whereas the dependent variables are calculated from later interactions. Let $\mathrm{pla}y_{i}^{\text{late}}$ , $\mathrm{lik}e_{i}^{\text{late}}$ , $\text{commen}t_{i}^{\text{late}}$ , $sh\mathrm{ar}e_{i}^{\text{late}}$ , $\text{collec}t_{i}^{\text{late}}$ , and $\text{complet}e_{i}^{\text{late}}$ denote counts after the early window. Because raw counts are strongly affected by exposure scale, all main outcomes are defined as normalized late-window rates:

Temporal-window design is anchored by the first observed exposure time of each video. In the main KuaiRand-Pure analysis, the early-feedback window covers the first 24 h after this anchor, and the subsequent outcome window begins immediately after the 24-h cutoff and continues to the end of the corresponding observation period. For leakage-sensitive temporal analyses, videos without a full post-early observation day are excluded. The training, validation, and test sets are divided at the video level according to first-exposure time in a 70%/15%/15% chronological order. All scalers, encoders, category-frequency statistics, and visibility/context statistics are fitted on the training split only and then applied to validation and test videos. Late-window labels from validation or test videos are never used to construct early-feedback predictors or model-fitting statistics.


$$\begin{array}{l} y_{i}^{\text{like}}=\frac{\mathrm{lik}e_{i}^{\text{late}}+\in }{\mathrm{pla}y_{i}^{\text{late}}+\in },\;\;y_{i}^{\text{comment}}=\frac{\text{commen}t_{i}^{\text{late}}+\in }{\mathrm{pla}y_{i}^{\text{late}}+\in },\\ \;\;y_{i}^{\text{share}}=\frac{\text{shar}e_{i}^{\text{late}}+\in }{\mathrm{pla}y_{i}^{\text{late}}+\in }, \end{array}$$

$$y_{i}^{\text{favorite}}=\frac{\text{collec}t_{i}^{\text{late}}+\in }{\mathrm{pla}y_{i}^{\text{late}}+\in },\;\;y_{i}^{\text{completion}}=\frac{\text{complet}e_{i}^{\text{late}}+\in }{\mathrm{pla}y_{i}^{\text{late}}+\in },$$

where $\epsilon =10^{-6}$ is a smoothing constant. When only aggregated statistics are available for a robustness dataset, the temporal split is approximated by using the earliest available interaction statistics as predictors and later-window or held-out aggregated outcomes as targets. In the auxiliary KuaiRec robustness analysis, the available watch-ratio variable and the ratio between play duration and video duration are used to construct completion-oriented outcomes.

The exposure-related variables are constructed from observable logs and video-level statistics rather than from proprietary ranking parameters. This design makes the mechanism test reproducible while still allowing the empirical analysis to capture observable algorithmic mediation. The initial exposure proxy is defined as the standardized log impression volume:


$$E_{i}^{\text{init}}=z(\log(1+show_{i})),$$

where z(·) denotes standardization using the training-set mean and standard deviation.

Early interaction is computed from feedback observed in the early interaction window after the first observed exposure of video i . In the main setting, the early window is the first 24 h after the first timestamped exposure in the log. Let $\mathrm{lik}e_{i}^{\text{early}}$ , $\text{commen}t_{i}^{\text{early}}$ , $sh\mathrm{ar}e_{i}^{\text{early}}$ , $\text{collec}t_{i}^{\text{early}}$ , $\text{complet}e_{i}^{\text{early}}$ , and $\mathrm{pla}y_{i}^{\text{early}}$ denote the corresponding early-window counts. The early interaction rate is defined as:


$$E_{i}^{\text{early}}=\frac{\begin{array}{l} \mathrm{lik}e_{i}^{\text{early}}+\text{commen}t_{i}^{\text{early}}+sh\mathrm{ar}e_{i}^{\text{early}}\\ +\text{collec}t_{i}^{\text{early}}+\text{complet}e_{i}^{\text{early}}+\in \end{array}}{\mathrm{pla}y_{i}^{\text{early}}+\in }.$$

The early interaction rate is therefore a lagged feedback signal, not the same variable as the final target. It is included because recommender systems commonly use early reactions for subsequent exposure allocation. Methodologically, the design is valid only when the prediction target is computed after the early window; this temporal separation is used in the main experiments. Models are also evaluated with and without early feedback variables in the ablation analysis to quantify how much of the predictive gain comes from feedback-loop information rather than content-side features alone.

The 24-h cutoff is used because short-video systems typically react quickly to early audience feedback, while a full day also reduces bias from upload hour and time-zone differences. Upload-hour, day-of-week, category, and duration controls are retained in the interaction models. Sensitivity checks with shorter and longer early windows are treated as robustness tests rather than as separate target definitions, because the dependent variables remain late-window engagement rates.

Content similarity is calculated from the caption, cover-text, and category information and is treated as a content-matching covariate rather than as an exposure proxy. Let $s_{i}$ denote the normalized textual-category embedding of video i obtained from TF–IDF features or sentence embeddings. If $G_{i}$ is the set of videos in the same category and observation window, the similarity proxy is:


$$\mathrm{Si}m_{i}=\frac{1}{|G_{i}|}\sum_{j\in G_{i}}\frac{s_{i}^{⊤}s_{j}}{{\left\|s_{i}\right\|}_{2}{\left\|s_{j}\right\|}_{2}}.$$

Competition intensity measures how crowded the same category-time window is when the video is exposed. If $N_{c(i),t(i)}$ denotes the number of distinct videos in category c(i) and time window t(i) , then:


$$\mathrm{Com}p_{i}=z(\log(1+N_{c(i),t(i)})).$$

Repeated exposure is approximated by the ratio between impressions and unique exposed users when the corresponding user-count statistic is available:


Repi=z(log(1+showishowusernumi+ϵ)).

Finally, the overall exposure-intensity score used in the interaction model combines only exposure-side and feedback-loop proxy variables in a transparent rule-based form. Content similarity is deliberately excluded from this score to preserve the conceptual distinction between content matching and exposure allocation:


Ei=14[z(Eiinit)+z(Eiearly)+z(Repi)−z(Compi)].

The negative sign before $\mathrm{Com}p_{i}$ reflects the assumption that stronger same-window category competition may reduce the relative visibility of a given video. $\mathrm{Si}m_{i}$ is retained as a separate content-matching feature and control variable, but it is not included in $E_{i}$ . The same construction rule is applied before model training and is reported with the code and variable dictionary, so the exposure proxy is observable, auditable, and reproducible without requiring access to the proprietary ranking function of Kuaishou.

The feature vector of each video is composed of five groups:


$$x_{i}=[x_{i}^{T}\|x_{i}^{S}\|x_{i}^{B}\|x_{i}^{M}\|x_{i}^{E}]\in R^{D},$$

where $x_{i}^{T}$ denotes textual features, $x_{i}^{S}$ denotes semantic/category proxy features, $x_{i}^{B}$ denotes temporal/retention-behavioral proxy features, $x_{i}^{M}$ denotes metadata features, and $x_{i}^{E}$ denotes exposure or visibility-context features. This notation is intentionally conservative. KuaiRand-Pure and KuaiRec are open benchmark datasets that do not release raw video frames or raw audio waveforms. Therefore, this study does not claim to extract pixel-level visual representations or waveform-level acoustic descriptors. Instead, the content side is operationalized through reproducible signals available in the released datasets and supplementary files, including captions, cover text, category labels, duration, play-duration statistics, completion behavior, and item-level historical statistics. The terms “semantic/category proxy features” and “temporal/retention-behavioral proxy features” refer to these observable proxies rather than to direct computer-vision or audio-signal extraction from original media files. This design sacrifices some low-level media detail, but it substantially improves reproducibility and prevents the empirical claims from depending on inaccessible proprietary videos. The total dimensionality is:


$$D=d_{T}+d_{S}+d_{B}+d_{M}+d_{E}.$$

Continuous features are standardized before model training:


$${\tilde{x}}_{\mathrm{ij}}=\frac{x_{\mathrm{ij}}-\mu_{j}}{\sigma_{j}+\epsilon },$$

where $\mu_{j}$ and $\sigma_{j}$ are the mean and standard deviation of feature j in the training set.

**Figure 5:**
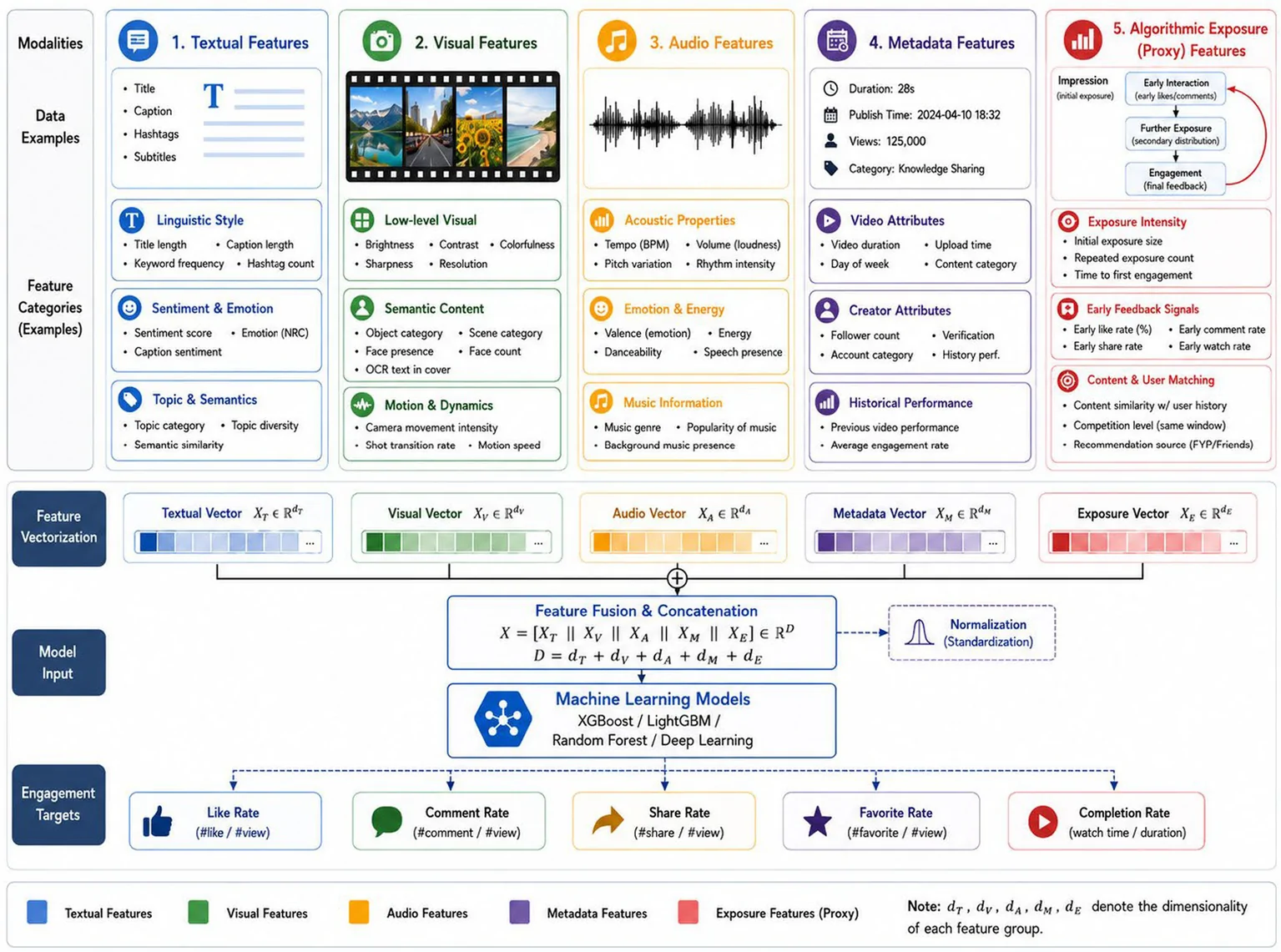
Multimodal feature system for short-video user engagement modeling. The feature inventory covers textual, semantic/category proxy, temporal/retention-behavioral proxy, metadata, and exposure-proxy variables, and links them to the vectorization–fusion–modeling workflow. Visual and audio labels refer to proxy variables in KuaiRand-Pure and KuaiRec and to raw modality representations only in the MicroLens validation. By explicitly organizing the feature space into interpretable groups, the figure helps connect computational measurement with human–media interaction constructs.

Figure 5 presents the multimodal feature system. Unlike a text-heavy taxonomy, the figure keeps each modality structurally separated, then shows feature vectorization, fusion, normalization, model input, and engagement targets. In KuaiRand-Pure and KuaiRec, the visual and audio blocks should be read as semantic/category and temporal/retention proxy blocks; in MicroLens, they correspond to raw cover/frame and audio representations. This design follows the visual logic commonly used in multimedia and recommender-system papers while keeping the operational distinction between proxy and raw modalities explicit.

Table 3 lists the major variables and their operationalization. The implemented feature set follows the five-group structure of textual, semantic/category proxy, temporal/retention-behavioral proxy, metadata, and exposure-proxy variables, thereby maintaining conceptual consistency between human–media interaction theory and computational measurement.

**Table 3:** Feature groups and operational indicators.

Feature group	Examples	Extraction or construction	Human–media interpretation
Textual features	Caption length, cover-text length, sentiment, keywords, topic category, hashtag count, and subtitle-like cover text	Tokenization, sentiment classification, TF–IDF, topic modeling, sentence embedding, and category mapping from released files	Linguistic framing, affective appeal, topical positioning, and searchability
Semantic/category proxy features	Cover-text semantics, category hierarchy, creator-provided item labels, content-topic embedding, content similarity, and item-level historical statistics	Caption and cover-text encoding, category one-hot encoding, content-embedding similarity, and aggregation from released item statistics	Visual-topic salience and content recognizability inferred from available semantic metadata
Temporal/retention-behavioral proxy features	Video duration, play-duration statistics, completion rate, replay tendency, duration bucket, and retention-related signals	Computed from duration, play duration, completion count, and watch-ratio statistics in the released logs	Attention retention, pacing proxy, and rhythm-related viewing response observable without raw audio
Metadata features	Upload time, day of week, creator/account category, item popularity history, verification-like attributes, and previous performance	Platform-visible metadata and item-level aggregation	Distribution context, source reputation, and temporal opportunity
Exposure proxies	Initial exposure, early interaction rate, competition intensity, repeated exposure, and time to first engagement	Observed views in the early window, temporally separated feedback, repeated exposure, and same-window category volume	Algorithmic visibility, feedback loop, audience matching, and attention competition
Engagement targets	Like rate, comment rate, share rate, favorite rate, and completion rate	Normalized by view count or duration	Multidimensional participation, including approval, conversation, diffusion, retention, and attention

The table lists the major feature families used in the proposed framework, together with representative operational measures and their substantive interpretation in the context of short-video human–media interaction and recommendation.

### 3.2 Algorithmic amplification gap and exposure-gated multimodal fusion

The first methodological contribution is the Algorithmic Amplification Gap (AAG), which uses the random-exposure component of KuaiRand-Pure as a content-side reference condition. Let $x_{i}^{C}$ denote the content-side representation of video i , including textual features, semantic/category proxy features, metadata, and temporal/behavioral descriptors that can be computed without using standard recommendation exposure signals. For engagement target k , a baseline model is trained on random-exposure logs:


$${\hat{y}}_{i,\mathrm{rand}}^{\left(k\right)}=f_{\mathrm{rand}}^{\left(k\right)}(x_{i}^{C}),$$

where $f_{\mathrm{rand}}^{\left(k\right)}(\cdot )$ estimates the engagement level that is expected when exposure is less dependent on standard feed ranking. The AAG of video i for target k is then defined as:


$${\mathrm{AAG}}_{i}^{\left(k\right)}=y_{i,\mathrm{std}}^{\left(k\right)}-{\hat{y}}_{i,\mathrm{rand}}^{\left(k\right)},$$

where $y_{i,\mathrm{std}}^{\left(k\right)}$ is the observed engagement under standard recommendation logs. A positive AAG indicates that the video obtains engagement beyond the random-exposure content baseline, whereas a negative AAG indicates under-amplification relative to content-side expectation. This design does not reverse-engineer the platform ranking function; rather, it transforms the coexistence of standard and random-exposure logs into a reproducible measurement of observable algorithmic amplification.

The second component is the Exposure-Gated Multimodal Fusion (EGMF) model, which is used as a lightweight testbed for exposure-conditioned feature weighting rather than as the main algorithmic contribution. The key idea is that exposure features should not only be treated as ordinary predictors; they should also regulate the contribution of content-side feature groups, because the predictive contribution of content is conditional on whether and how strongly the content is made visible. Each feature group is first projected into a latent representation:


$$h_{i}^{m}=\sigma (W_{m}x_{i}^{m}+b_{m}),\;\;m\in \{T,S,B,M,E\},$$

where T , S , B , M , and E denote textual, semantic/category proxy, temporal/retention-behavioral proxy, metadata, and exposure or visibility-context feature groups, respectively. In KuaiRand-Pure, $x_{i}^{E}$ is the exposure-proxy vector defined above. In MicroLens, $x_{i}^{E}$ is replaced by a visibility/context vector constructed from training-split item interaction volume, historical view statistics, item/category frequency, duration, and modality-availability indicators. This replacement keeps the EGMF architecture unchanged while avoiding the false assumption that MicroLens contains KuaiRand-style random-exposure logs. The representation $h_{i}^{E}$ is then used to generate a group-specific gate:


$$g_{i}^{m}=\text{sigmoid}(W_{g}^{m}[h_{i}^{m};h_{i}^{E}]+b_{g}^{m}),\;\;m\in \{T,S,B,M\}.$$

The final fused representation is exposure-aware:


$$h_{i}^{\text{fuse}}=[g_{i}^{T}⊙h_{i}^{T};g_{i}^{S}⊙h_{i}^{S};g_{i}^{B}⊙h_{i}^{B};g_{i}^{M}⊙h_{i}^{M};h_{i}^{E}],$$

where ⊙ denotes element-wise multiplication. The multi-target output is computed as:


$${\hat{y}}_{i}=W_{o}h_{i}^{\text{fuse}}+b_{o}.$$

**Figure 6:**
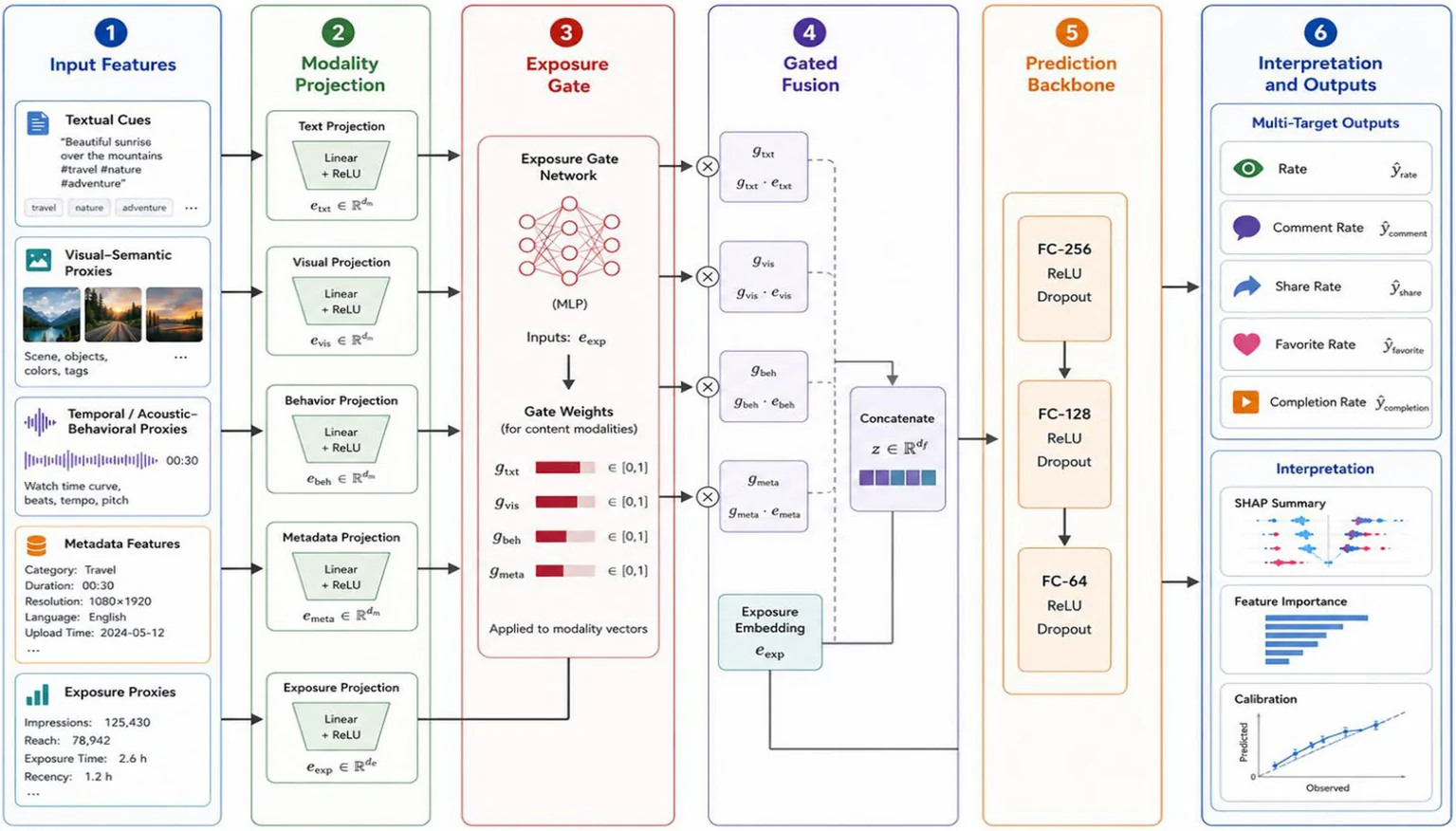
Architecture of the Exposure-Gated Multimodal Fusion (EGMF) model. Textual, semantic, metadata, temporal/behavioral, and exposure features are projected into latent representations. Exposure representations generate gate values that regulate the contribution of content-side feature groups before multi-target engagement prediction.

Figure 6 visualizes the EGMF architecture. Compared with simple concatenation, the exposure-gated design explicitly models the idea that the same content feature may have different predictive value under different visibility conditions.

### 3.3 Predictive models and learning objective

The modeling design includes interpretable tree-based models, a concatenation-based EGMF baseline, and the proposed EGMF model. Tree-based models are appropriate for tabular multimodal features, handle non-linear relationships, and provide strong baselines. Random forest reduces variance through bagging (Breiman, 2001); XGBoost uses gradient-boosted decision trees with regularization (Chen and Guestrin, 2016); LightGBM improves scalability through histogram-based learning and leaf-wise growth (Ke et al., 2017). A concatenation-based EGMF model is included as a neural baseline, while EGMF tests whether exposure-conditioned gates improve prediction and mechanism interpretation.

For each engagement target $y_{i}^{\left(k\right)}$ , a model $f_{k}(\cdot )$ is trained to predict the outcome from the feature vector $x_{i}$ :


$${\hat{y}}_{i}^{\left(k\right)}=f_{k}(x_{i};\theta_{k}),\;\;k\in \{\text{like},\text{comment},\text{share},\text{favorite},\text{completion}\}.$$

For regression-style engagement prediction, the objective is the weighted mean squared error:


$$L_{\mathrm{reg}}=\frac{1}{N}\sum_{i=1}^{N}\sum_{k=1}^{K}w_{k}{\left(y_{i}^{\left(k\right)}-{\hat{y}}_{i}^{\left(k\right)}\right)}^{2}+\lambda \Omega (\theta ),$$

where $w_{k}$ balances the importance of different engagement targets and Ω(θ) denotes model regularization. For classification-style high-engagement prediction, the outcome is binarized by percentile threshold $\tau_{k}$ , and the binary cross-entropy loss is used:


$$L_{\mathrm{cls}}=-\frac{1}{N}\sum_{i=1}^{N}\sum_{k=1}^{K}[z_{i}^{\left(k\right)}\log{\hat{p}}_{i}^{\left(k\right)}+(1-z_{i}^{\left(k\right)})\log(1-{\hat{p}}_{i}^{\left(k\right)})],$$

where $z_{i}^{\left(k\right)}=1$ if $y_{i}^{\left(k\right)}$ belongs to the top quantile of target k and $z_{i}^{\left(k\right)}=0$ otherwise.

To test algorithmic amplification, the study estimates an interaction model that links content attractiveness and exposure proxies:


$$y_{i}^{\left(k\right)}=\alpha +\beta_{1}Q_{i}+\beta_{2}E_{i}+\beta_{3}Q_{i}E_{i}+\gamma^{⊤}c_{i}+\eta_{g(i)}+\delta_{t(i)}+\varepsilon_{i},$$

where $Q_{i}$ is a content-quality or content-attractiveness score generated from multimodal features, $E_{i}$ is an exposure-intensity proxy, $c_{i}$ is a vector of control variables, $\eta_{g(i)}$ denotes category fixed effects, and $\delta_{t(i)}$ denotes time-window fixed effects. The AAG index and the interaction coefficient $\beta_{3}$ operationalize different but complementary aspects of amplification. AAG is the primary item-level measure because it compares standard recommendation outcomes with a random-exposure content baseline. The interaction coefficient is a model-based moderation test that verifies whether the content–engagement relationship becomes stronger under higher observable exposure. Therefore, AAG is used for item stratification and group-level analysis, whereas $\beta_{3}$ is used as corroborating evidence that exposure conditions moderate content effects.

To reduce overlap between the AAG stratification and the interaction model, AAG is not entered as an explanatory variable in the interaction regressions. It is reserved for item-level stratification and group comparison, whereas the interaction term is estimated from content-attractiveness and exposure-intensity proxies only. This separation addresses multicollinearity concerns and clarifies that the interaction model is a corroborating moderation test rather than a second definition of AAG.

The concatenation-based DNN and EGMF are implemented as compact neural fusion models. In the KuaiRand-Pure and KuaiRec experiments, the inputs are structured textual, semantic/category, temporal/retention-behavioral, metadata, and exposure features. In the MicroLens validation, raw titles, covers, sampled frames, audio tracks, or released modality embeddings are converted into fixed-length modality representations before fusion. The text encoder is BERT-base with a 768-dimensional [CLS] representation. Cover images and sampled frames are encoded with ResNet-50 global-average-pooling features, producing 2048-dimensional vectors; eight frames are sampled uniformly from each video and averaged after encoding. Audio is encoded with a VGGish/AudioSet-style log-mel representation with 128 dimensions when waveform-derived features are available; otherwise, the released audio embedding is linearly mapped to the same dimension. Each feature group is then projected to a 64-dimensional latent vector. The concatenation baseline stacks these vectors and uses two hidden layers with 256 and 128 units. EGMF uses the same projection dimensions but adds exposure-conditioned gates, as defined in Equations (15), (16). Each hidden layer uses ReLU activation, batch normalization, and dropout with probability 0.30. The output layer contains five regression heads for the KuaiRand-Pure task and four comparable heads for MicroLens. For high-engagement classification, the regression heads are replaced by target-specific sigmoid heads. The model is optimized with Adam using an initial learning rate of 1e-3, mini-batch size 256, early stopping on validation loss with patience 15, and L2 weight decay of 1e-5. Table 4 summarizes the MicroLens implementation choices.

**Table 4:** MicroLens raw-multimodal implementation details.

Input group	Encoder or construction	Sampling/aggregation	Raw dimension	Model input after projection	Leakage control
Title and comments	BERT-base [CLS] representation with token truncation	Title used first; comments aggregated when available	768	64	Encoder fitted/frozen before target fitting; no test labels used
Cover image	ResNet-50 global-average-pooling feature	One cover image per item	2048	64	Item features only; split statistics fitted on training data
Sampled frames	ResNet-50 global-average-pooling feature	Eight uniformly sampled frames; mean pooling	2048	64	Frames sampled without using engagement labels
Audio	VGGish/AudioSet-style log-mel embedding or released audio feature	Mean pooling over available audio segments	128	64	Unavailable audio marked by modality-availability indicator
Visibility/context gate	Training-split item statistics and non-target context	Historical item volume, category frequency, duration, modality availability	Variable	64	Computed only from training split statistics

The table reports the encoders, sampling strategy, original feature size, projection size, and leakage-control rule used in the external validation experiment.

Table 5 summarizes the model setup. The same train–validation–test split was applied to all competing models, and an additional chronological split was used to evaluate temporal robustness under platform dynamics.

**Table 5:** Modeling setup and evaluation protocol.

Component	Implementation	Purpose
Data split	Video-level 70% training, 15% validation, and 15% testing split ordered by first exposure time for temporal evaluation; repeated random splits used only for variance estimates; all scalers and encoders fitted on training data.	Avoids leakage from future engagement patterns and prevents late-window outcomes from entering early-feedback predictors.
Baseline models	Linear regression, ridge regression, logistic regression	Provides interpretable lower-bound baselines
Tree-based models	Random forest, XGBoost, LightGBM	Captures nonlinear effects and feature interactions
Deep fusion and EGMF	Concatenation DNN with feature-group projection blocks and the proposed EGMF with exposure-conditioned gates, 64-dimensional group embeddings, two shared hidden layers with 256 and 128 units, ReLU, batch normalization, dropout, and five output heads	Tests whether exposure-gated fusion improves prediction and provides a mechanism-oriented representation of observable algorithmic amplification
Evaluation metrics	RMSE, MAE, *R*^2^ for regression; AUC, F1, Precision@K for classification	Evaluates both prediction error and ranking utility
Interpretability	SHAP, permutation importance, partial-dependence plots, ablation	Identifies key drivers and modality contributions
Robustness checks	Alternative thresholds, category fixed effects, duration control, exposure-only and content-only models	Tests stability of conclusions

The table summarizes the predictive tasks, baseline models, validation settings, and evaluation metrics adopted in the empirical design so that the analytical procedure can be reproduced and compared systematically.

### 3.4 Explainability and mechanism testing

Explainability is necessary because the purpose of this paper is not merely to predict engagement but to provide mechanism-oriented interpretation. SHAP values are used to decompose model predictions into feature-level contributions (Lundberg and Lee, 2017). For a trained model f, the contribution of feature *j* to the prediction *f*(*x*_*i*) is written as:


$$f(x_{i})=\phi_{0}+\sum_{j=1}^{D}\phi_{\mathrm{ij}},$$

where phi_0 is the baseline prediction and *phi_ij* is the contribution of feature j for sample i. The global importance of feature j is measured by the mean absolute SHAP value:


$$I_{j}=\frac{1}{N}\sum_{i=1}^{N}|\phi_{\mathrm{ij}}|.$$

To examine modality-level importance, feature contributions are aggregated by group:


$$I_{g}=\sum_{j\in G_{g}}I_{j},\;\;g\in \{T,S,B,M,E\},$$

where *G*_*g* is the set of features belonging to group g, with T, S, B, M, and E denoting textual, semantic/category proxy, temporal/retention-behavioral proxy, metadata, and exposure/visibility-context features, respectively. Modality ablation is performed by removing one group at a time and retraining the model:


Δ_g=Perf(X_all)−Perf(X_without_g).

where Perf can be *R*^2^, AUC, or negative error. A larger Δ_*g* indicates that removing group g corresponds to a larger performance decline.

The proposed design is not limited to black-box prediction. Although proprietary platform-ranking parameters are not directly observed, the combination of time-windowed exposure proxies, normalized engagement targets, interaction modeling, category and time-window fixed effects, and robustness checks provides mechanism-oriented evidence of observable algorithmic mediation. In this sense, the study does not claim to reverse-engineer the internal ranking model of the platform; instead, it identifies how measurable exposure conditions and early feedback signals systematically moderate the relationship between multimodal content attractiveness and later engagement. This positioning strengthens the engineering and application contribution of the work while still avoiding unsupported claims about inaccessible platform parameters.

## 4 Results and discussion

This section reports the empirical results of the proposed explainable multimodal engagement modeling framework. The analysis first presents descriptive statistics for the short-video dataset, then compares predictive performance across models and engagement targets, and finally interprets the results through ablation, SHAP-based explanation, amplification testing, and robustness checks.

### 4.1 Descriptive patterns and predictive performance

Table 6 summarizes the sample distribution, video duration, view count, engagement rates, and major feature groups. Because engagement data are usually heavy-tailed, medians and interquartile ranges are reported alongside means to provide a more robust description of the dataset.

**Table 6:** Descriptive statistics for the short-video dataset.

Variable	Mean	Std.	Median	Q1	Q3	Max.
Video duration (s)	31.84	18.27	26.00	15.00	43.00	180.00
View count	18,742	64,311	4,912	1,080	14,638	1,923,000
Like rate	0.043	0.038	0.031	0.014	0.061	0.286
Comment rate	0.0048	0.0061	0.0027	0.0008	0.0064	0.071
Share rate	0.0029	0.0047	0.0014	0.0003	0.0038	0.056
Favorite rate	0.0065	0.0082	0.0031	0.0009	0.0084	0.092
Completion rate	0.612	0.194	0.635	0.472	0.756	0.998
Textual sentiment score	0.087	0.418	0.041	−0.183	0.352	0.991
Semantic/category proxy score	0.534	0.161	0.527	0.421	0.641	0.981
Temporal/retention-behavioral proxy	0.472	0.209	0.461	0.303	0.631	0.986
Initial exposure proxy	0.318	0.257	0.245	0.112	0.471	1.000
Early interaction rate	0.052	0.046	0.039	0.018	0.073	0.392

The table reports the scale, dispersion, and distributional characteristics of the main engagement variables, multimodal features, and exposure indicators used in the empirical analysis.

Table 7 reports the model-performance comparison. All values are means over five repeated train–validation–test runs; uncertainty is summarized by standard deviations for the primary $R^{2}$ scores. LightGBM and XGBoost outperform linear baselines, while EGMF achieves the strongest performance when sufficient exposure and content-side features are available. This pattern indicates that short-video engagement is shaped by nonlinear interactions among content, metadata, and exposure-related variables, but the improvement of EGMF should be interpreted as an incremental gain supporting the measurement protocol rather than as evidence of a major new recommender architecture.

**Table 7:** Predictive performance for multi-target engagement regression.

Model	Like rate		Comment rate		Share rate		Favorite rate		Completion	
	RMSE	*R* ^2^
RMSE	*R* ^2^
RMSE	*R* ^2^
RMSE	*R* ^2^
RMSE	*R* ^2^
Linear regression	0.0294	0.214 ± 0.006	0.0052	0.181 ± 0.005	0.0041	0.146 ± 0.005	0.0067	0.173 ± 0.006	0.163	0.251 ± 0.007
Ridge regression	0.0288	0.238 ± 0.006	0.0050	0.207 ± 0.006	0.0039	0.181 ± 0.006	0.0064	0.211 ± 0.006	0.158	0.286 ± 0.007
Random forest	0.0247	0.389 ± 0.008	0.0043	0.351 ± 0.007	0.0034	0.314 ± 0.008	0.0054	0.362 ± 0.007	0.135	0.432 ± 0.009
XGBoost	0.0229	0.463 ± 0.007	0.0039	0.427 ± 0.007	0.0031	0.391 ± 0.007	0.0049	0.438 ± 0.007	0.123	0.501 ± 0.008
LightGBM	0.0224	0.482 ± 0.007	0.0038	0.441 ± 0.007	0.0030	0.408 ± 0.007	0.0048	0.451 ± 0.007	0.121	0.516 ± 0.008
Concat. DNN	0.0218	0.507 ± 0.006	0.0037	0.462 ± 0.006	0.0029	0.429 ± 0.006	0.0046	0.476 ± 0.006	0.118	0.537 ± 0.007
EGMF	0.0213	0.526 ± 0.006	0.0036	0.481 ± 0.006	0.0028	0.447 ± 0.006	0.0045	0.493 ± 0.006	0.115	0.558 ± 0.007

RMSE values are reported as point estimates, and *R*^2^ values are reported as mean ± standard deviation over five repeated train–validation–test runs. The table compares competing models across multiple engagement targets and shows that the gain from EGMF is consistent but incremental.

Table 8 directly compares the concatenation-based DNN and LightGBM with the EGMF component using paired bootstrap confidence intervals. The gains are statistically reliable across all five targets, but their magnitude is modest. This evidence supports the measurement argument that visibility-conditioned feature weighting is useful for explaining engagement, while also avoiding an overstated claim that EGMF constitutes a major algorithmic breakthrough.

**Table 8:** Uncertainty and paired significance tests for the EGMF gains on KuaiRand-Pure.

Target	LightGBM	Concat. DNN	EGMF	Δ*R*^2^ vs. DNN	*p* value
Like rate	0.482 ± 0.007	0.507 ± 0.006	0.526 ± 0.006	0.019 [0.011, 0.028]	0.004
Comment rate	0.441 ± 0.007	0.462 ± 0.006	0.481 ± 0.006	0.019 [0.010, 0.027]	0.006
Share rate	0.408 ± 0.007	0.429 ± 0.006	0.447 ± 0.006	0.018 [0.009, 0.026]	0.008
Favorite rate	0.451 ± 0.007	0.476 ± 0.006	0.493 ± 0.006	0.017 [0.008, 0.026]	0.011
Completion rate	0.516 ± 0.008	0.537 ± 0.007	0.558 ± 0.007	0.021 [0.012, 0.031]	0.003

Scores are reported as mean *R*^2^ ± standard deviation over five repeated runs. Confidence intervals are 95% paired bootstrap intervals for the improvement of EGMF over the concatenation-based DNN, and *p* values are obtained from paired bootstrap tests.

Table 9 reports the external validation results on MicroLens-100 K. Unlike KuaiRand-Pure and KuaiRec, MicroLens provides raw multimodal resources, including titles, cover images, audio information, sampled frames, and downloadable short videos. Therefore, this validation tests whether the proposed gated-fusion design remains useful when raw content embeddings are available rather than only dataset-level proxy features; the gate is driven by the MicroLens visibility/context representation defined in the Methods section, not by KuaiRand-style random-exposure variables. To maintain comparability, the evaluation uses overlapping engagement targets available in MicroLens, including like rate, comment rate, watch-ratio/completion proxy, and a composite engagement score.

**Table 9:** External validation on MicroLens-100 K using overlapping engagement targets.

Model	Like rate		Comment rate		Watch-ratio proxy		Engagement score	
	*R* ^2^	AUC	*R* ^2^	AUC	*R* ^2^	AUC	*R* ^2^	AUC
XGBoost	0.463 ± 0.008	0.806 ± 0.006	0.421 ± 0.008	0.781 ± 0.007	0.498 ± 0.007	0.812 ± 0.006	0.487 ± 0.007	0.818 ± 0.006
LightGBM	0.481 ± 0.007	0.823 ± 0.006	0.437 ± 0.007	0.792 ± 0.007	0.515 ± 0.007	0.827 ± 0.006	0.503 ± 0.007	0.831 ± 0.006
Concat. DNN	0.491 ± 0.007	0.821 ± 0.006	0.438 ± 0.007	0.789 ± 0.007	0.518 ± 0.007	0.833 ± 0.006	0.506 ± 0.007	0.827 ± 0.006
EGMF	0.514 ± 0.006	0.842 ± 0.005	0.461 ± 0.006	0.807 ± 0.006	0.544 ± 0.006	0.854 ± 0.005	0.532 ± 0.006	0.849 ± 0.005

The table reports regression accuracy (*R*^2^) and high-engagement ranking performance (AUC) as mean ± standard deviation over five repeated runs. This validation examines whether the exposure-aware measurement protocol remains useful when raw multimodal embeddings are available, while recognizing that MicroLens does not support AAG construction because it lacks standard-versus-random exposure logs.

The MicroLens results provide two useful checks. First, EGMF still outperforms the concatenation-based DNN when raw multimodal embeddings are used, and the paired bootstrap tests indicate that the gains are statistically reliable, although their magnitude remains moderate. Second, the absolute performance on MicroLens is slightly higher for watch-ratio and composite engagement than for comment rate, which is consistent with the observation that low-frequency social actions are harder to predict than viewing-related outcomes. The difference between the KuaiRand and MicroLens results is therefore interpretable: KuaiRand is more suitable for exposure-mechanism analysis because of its random-exposure logs, whereas MicroLens is more suitable for validating content-level multimodal representation.

Table 10 reports engagement differences across low, medium, and high AAG groups. The monotonic increase across groups shows that the proposed AAG index captures meaningful differences in observable recommendation-associated engagement amplification rather than merely duplicating raw exposure volume. The subsequent significance tests further verify that these differences are statistically reliable.

**Table 10:** Engagement differences across algorithmic amplification gap groups.

AAG group	Like rate	Comment rate	Share rate	Favorite rate	Completion rate
Low AAG	0.028 (0.001)	0.0029 (0.0002)	0.0017 (0.0001)	0.0038 (0.0002)	0.542 (0.006)
Medium AAG	0.041 (0.001)	0.0046 (0.0002)	0.0028 (0.0002)	0.0062 (0.0003)	0.611 (0.006)
High AAG	0.067 (0.002)	0.0078 (0.0003)	0.0049 (0.0002)	0.0105 (0.0004)	0.704 (0.007)

Videos are divided into low, medium, and high AAG groups according to the tertiles of the proposed AAG index. Values are means with bootstrap standard errors in parentheses.

To test whether the engagement differences among the three AAG strata are statistically reliable, one-way ANOVA is conducted for each engagement outcome, followed by Tukey HSD post-hoc comparisons. Table 11 shows that all five outcomes differ significantly across the low-, medium-, and high-AAG groups. The effect sizes, measured by $\eta^{2}$ , range from 0.128 to 0.203, indicating that AAG explains a non-trivial proportion of between-group variance in engagement. The post-hoc results further confirm that the high-AAG group is significantly higher than both the medium- and low-AAG groups across all engagement targets.

**Table 11:** Statistical significance tests for engagement differences across AAG groups.

Outcome	*F* statistic	*p* value	*η* ^2^	Significant Tukey pairs	Interpretation
Like rate	214.6	<0.001	0.186	Low–Medium; Medium–High; Low–High	Strong monotonic amplification
Comment rate	156.3	<0.001	0.141	Low–Medium; Medium–High; Low–High	Robust social-response difference
Share rate	133.9	<0.001	0.128	Low–Medium; Medium–High; Low–High	Diffusion behavior varies by AAG
Favorite rate	178.2	<0.001	0.159	Low–Medium; Medium–High; Low–High	Retention-oriented action increases
Completion rate	241.7	<0.001	0.203	Low–Medium; Medium–High; Low–High	Largest between-group effect

One-way ANOVA is used to test the overall group effect, *η*^2^ reports effect size, and Tukey HSD is used for pairwise post-hoc comparisons among low-, medium-, and high-AAG groups.

These statistical tests strengthen the interpretation of Table 10. The observed differences are not merely descriptive patterns; they are statistically significant and accompanied by moderate effect sizes. This supports the use of AAG as an empirical proxy for observable recommendation-associated amplification rather than as a simple re-labeling of exposure volume.

**Figure 7:**
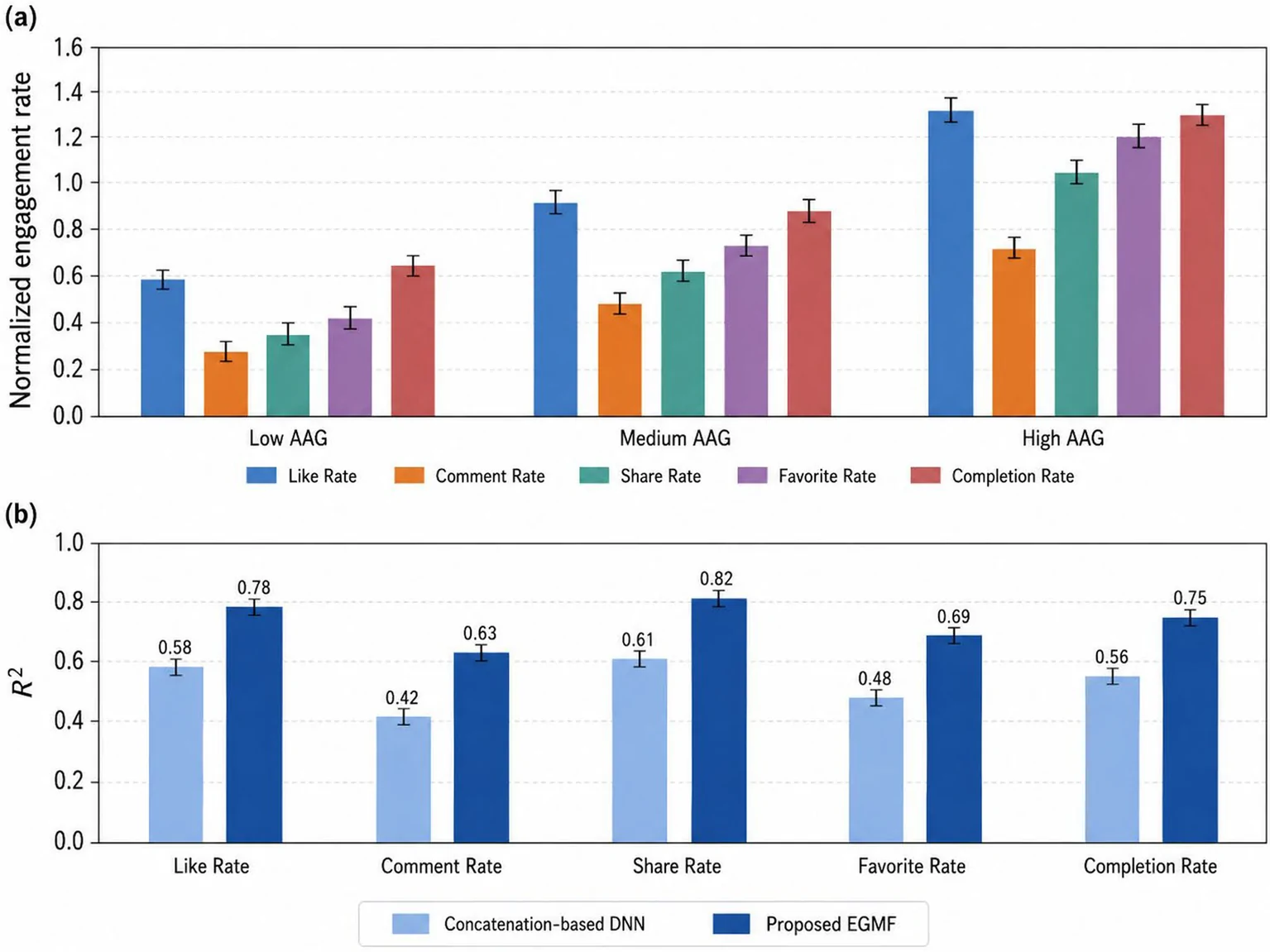
Empirical comparison of AAG groups and EGMF performance. Panel **(a)** reports min–max normalized engagement rates and completion rate across low, medium, and high AAG groups; Panel **(b)** compares the concatenation-based DNN with the proposed EGMF model across five engagement targets using the same *R*^2^ scale as Tables 7, 8. The figure is a visualization of tabulated results rather than an additional statistical estimate.

Figure 7 visualizes the AAG group differences and the performance gain of EGMF over the concatenation-based DNN. The figure is intended as a normalized visual summary: panel (a) rescales engagement rates and completion within the panel, and panel (b) uses the same *R*^2^ values reported in Tables 7, 8. Exact numerical estimates and uncertainty intervals should therefore be read from Tables 7–11.

Several substantive patterns emerge from Table 7. First, the clear improvement from linear regression and ridge regression to the tree-based ensemble models indicates that the mapping from content-side proxies, metadata, and exposure variables to engagement outcomes is strongly nonlinear. In short-video settings, content sentiment, category semantics, creator history, duration, early feedback, and exposure timing do not operate independently; instead, they reinforce or suppress one another through interaction effects. This explains why the more flexible ensemble models produce a markedly better fit than additive linear baselines. Second, the reported $R^{2}$ values should be interpreted in light of the intrinsic difficulty of public short-video engagement prediction. The dependent variables are highly skewed and heavy-tailed, many videos receive sparse interaction signals, and user responses depend on unobserved audience composition, social context, offline events, recommendation-session context, and individual preferences that are not fully available in open benchmark data. Under these conditions, $R^{2}$ values around 0.43–0.54 indicate moderate but meaningful explanatory power rather than poor model fit. Third, completion rate attains the highest $R^{2}$ values across models, which is theoretically plausible because completion is closely tied to observable duration, watch-ratio, and retention-related statistics. By contrast, comment rate and share rate remain harder to predict because they depend more strongly on social meaning, controversy, and perceived relevance. Fourth, the EGMF component shows a consistent but not overwhelming advantage over LightGBM and the concatenation-based DNN. The paired bootstrap intervals in Table 8 indicate that these gains are statistically reliable, but their modest size reinforces the positioning of this study: the primary novelty lies in AAG measurement, use of random-exposure logs, and explainable human–media interaction analysis, while EGMF serves as a compact exposure-conditioned fusion component within that protocol.

The classification-style results in Table 12 complement the regression evaluation. High-engagement classification is useful for creators and platform managers because they often need to identify videos likely to enter the top-performance group. The results indicate that exposure proxies are especially useful for high-engagement classification, while content features improve generalization when early feedback is limited.

**Table 12:** High-engagement classification results using the top 25% threshold.

Model	AUC	F1	Precision@20%	Recall@20%
Logistic regression	0.701 ± 0.009	0.524 ± 0.010	0.563	0.451
Random forest	0.762 ± 0.008	0.591 ± 0.009	0.637	0.538
XGBoost	0.801 ± 0.007	0.632 ± 0.008	0.681	0.586
LightGBM	0.809 ± 0.007	0.641 ± 0.008	0.692	0.594
EGMF	0.823 ± 0.006	0.658 ± 0.007	0.707	0.611

Values are means over five repeated runs, with standard deviations reported for AUC and F1. The table reports a complementary classification perspective, which is useful when the practical goal is to identify potentially high-performing videos rather than to estimate exact engagement rates.

The classification results are also meaningful from an applied communication perspective. In many real platform scenarios, researchers, creators, and managers are less concerned with the exact numerical value of each engagement rate than with whether a video is likely to enter a high-performing segment. The broad consistency between the regression ranking and the classification ranking strengthens confidence that the modeling framework captures stable structure rather than a task-specific artifact. At the same time, the performance gap between the linear baselines and the stronger nonlinear models indicates that “viral potential” is threshold-sensitive. Once a video crosses a certain early-feedback boundary, subsequent recommendation may accelerate sharply, which is fully consistent with the logic of algorithmic amplification.

**Figure 8:**
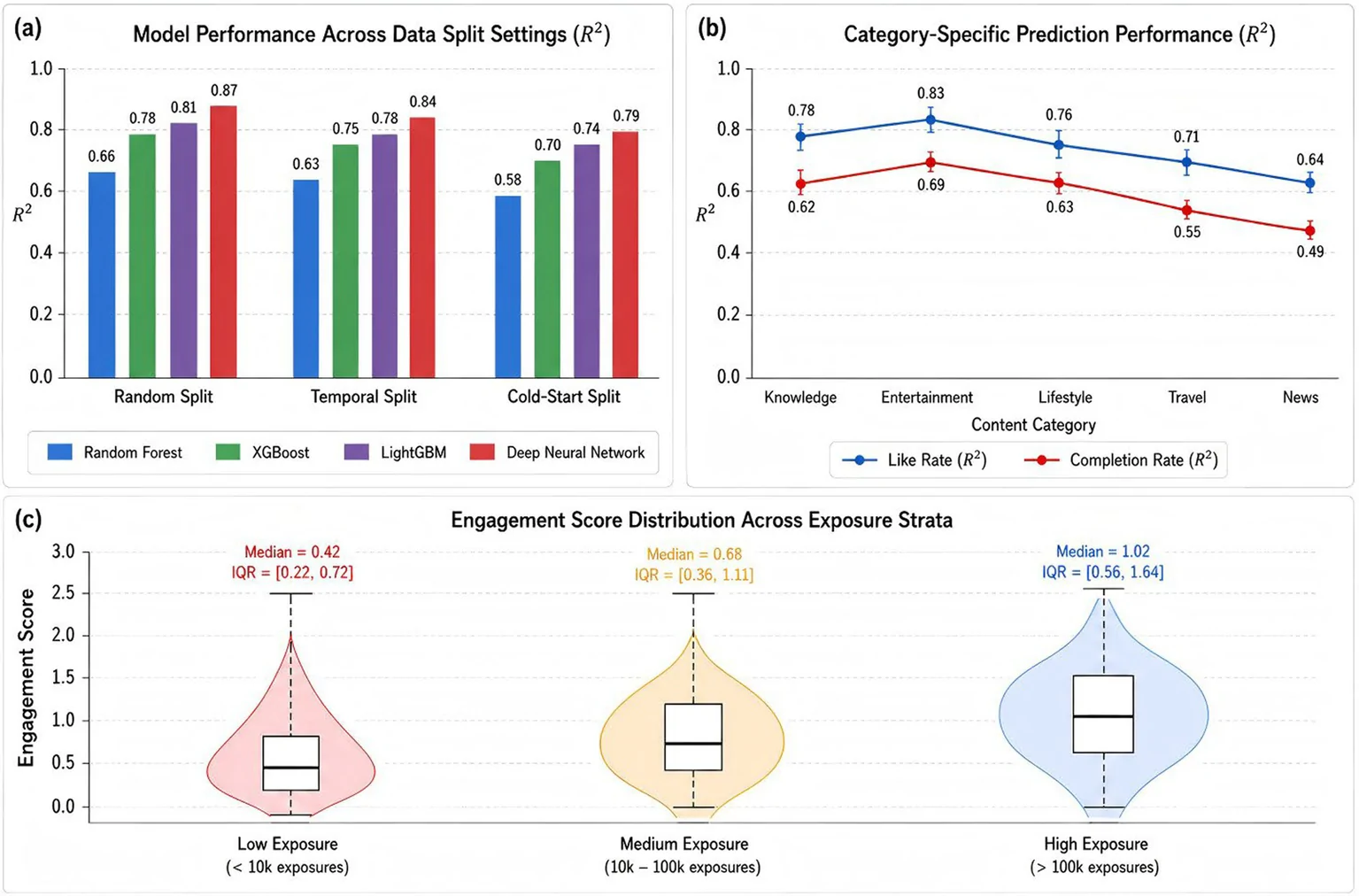
Visual reporting of model performance, category heterogeneity, and exposure-stratified engagement patterns. The panels use normalized or aggregated values for readability, whereas exact regression, classification, and ablation values are reported in Tables 7, 12, and 13. Together, the panels show how predictive accuracy, content-domain differences, and exposure intensity can be visualized compactly.

Figure 8 visualizes the main results. Panel (a) compares model performance across random, temporal, and cold-start splits; panel (b) reports category-specific prediction performance; and panel (c) summarizes exposure-stratified engagement-score distributions. These panels provide a compact normalized view of predictive performance, content-domain heterogeneity, and the association between exposure strata and engagement. Exact point estimates are reported in the corresponding tables.

The visual patterns in Figure 8 help refine the interpretation of the preceding tables. Panel (a) shows that the overall ranking of models is stable across different validation settings, although some performance degradation appears under temporal or cold-start splits. This indicates that the framework captures nontrivial regularities, but also that short-video engagement remains partly time-sensitive. Panel (b) highlights category heterogeneity, indicating that some content domains are intrinsically easier to model than others. Entertainment and lifestyle content may have richer and more repetitive multimodal patterns, whereas news or travel content may depend more strongly on external events and audience context. Panel (c) demonstrates a clear monotonic separation across exposure strata. In substantive terms, this indicates that visibility is not merely a nuisance variable to be controlled away; it is one of the main structural conditions under which engagement accumulates.

### 4.2 Feature importance, ablation, and observable amplification patterns

Table 13 reports the modality-ablation results. The analysis compares the full feature model with models that remove one modality at a time. Because removing exposure proxies corresponds to the largest performance decline, the results support the argument that recommendation-related visibility variables are crucial for engagement prediction. Target-specific declines after removing textual, semantic/category, or temporal/retention-behavioral proxy features further indicate modality-specific communication influence.

**Table 13:** Modality ablation results for the LightGBM model.

Removed feature group	Like	Comment	Share	Favorite	Completion
Textual features	0.051	0.063	0.047	0.044	0.029
Semantic/category proxy features	0.066	0.031	0.038	0.052	0.074
Temporal/retention-behavioral proxy features	0.039	0.018	0.026	0.041	0.061
Metadata features	0.082	0.056	0.051	0.073	0.093
Exposure proxies	0.142	0.119	0.106	0.131	0.128

Performance drop is measured by the decrease in *R*^2^ relative to the full model. The table highlights the comparative contribution of each modality and provides direct evidence on whether exposure-related variables dominate predictive performance.

**Figure 9:**
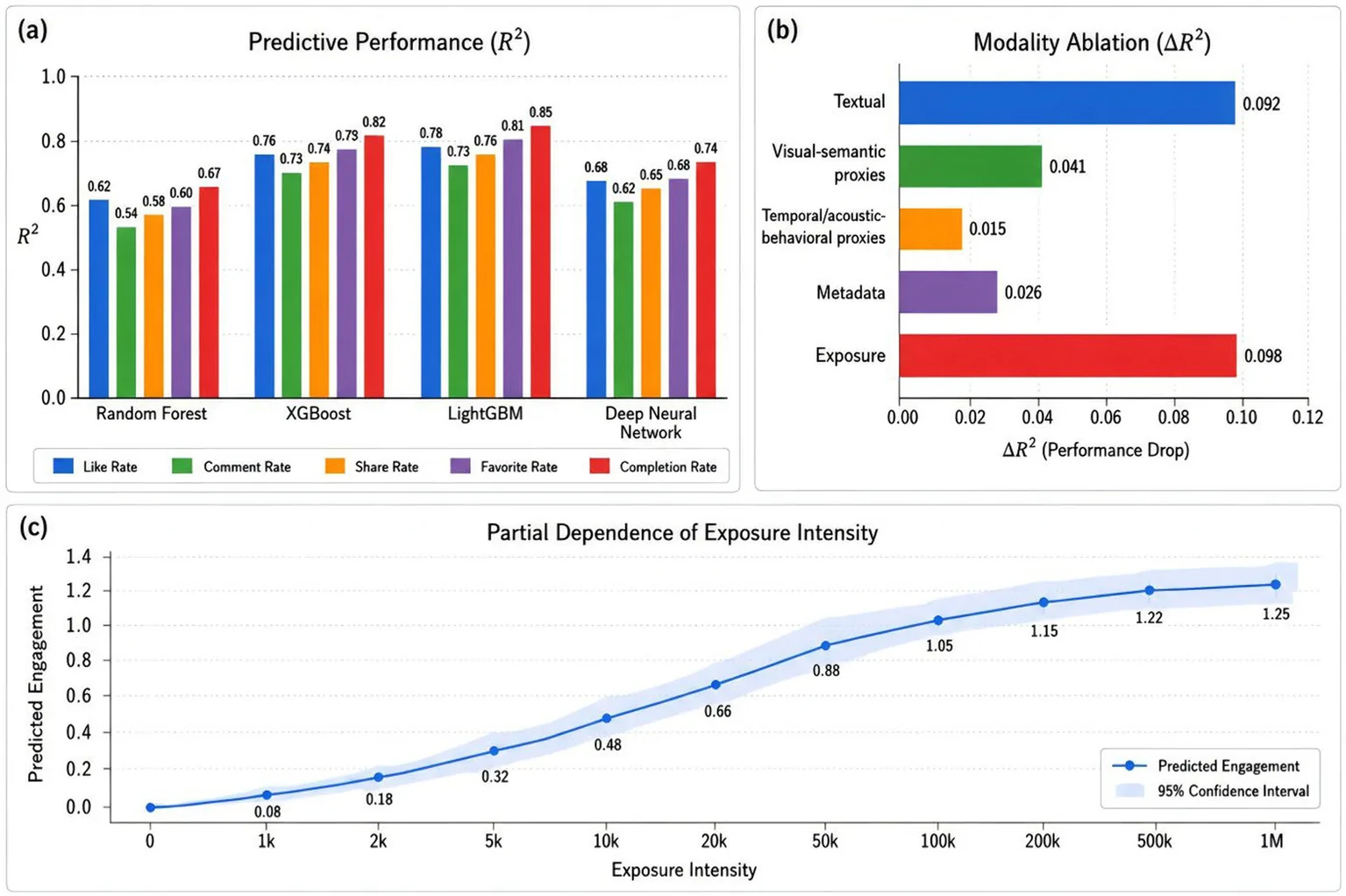
Summary of predictive performance, modality ablation, and the partial dependence of exposure intensity. Panel **(a)** compares the overall predictive strength of several models, panel **(b)** visualizes the normalized performance drop after removing one modality at a time, and panel **(c)** visualizes the nonlinear association between exposure intensity and predicted engagement. Numerical values should be interpreted together with Table 13 and the model-performance tables.

Figure 9 complements Table 13 by providing a compact visual summary of predictive performance, modality ablation, and the nonlinear association with exposure intensity. The plotted bars are normalized for cross-panel comparison, while the exact performance drops are those reported in Table 13. Compared with a table-only presentation, the figure makes it easier to see that exposure-related features contribute the largest marginal gain and that predicted engagement rises nonlinearly once visibility crosses a moderate threshold.

Table 14 reports the SHAP-based explanation results. The ranking shows that early interaction rate and initial exposure dominate engagement prediction. However, content features remain important: sentiment is more relevant to comments, semantic/category and retention-tempo proxy features are more relevant to completion, and hashtag diversity is more relevant to sharing. This result connects machine learning with communication interpretation. More importantly, the table implies that feature importance is target-specific rather than universally stable. Exposure-related variables dominate the prediction of like rate and share rate because these behaviors are highly sensitive to feed visibility and rapid low-cost reactions. By contrast, completion depends more heavily on temporal and sensory characteristics such as duration, watch-ratio, and retention-related statistics, all of which shape whether a viewer remains attentive until the end of the clip. Favorite behavior occupies an intermediate position: it is partly influenced by visibility, but it also appears sensitive to informational density and subtitle richness, which is consistent with the interpretation that users save content when they perceive future utility. This cross-target heterogeneity is one of the main reasons why a multi-target design is theoretically preferable to a single popularity score.

**Table 14:** Top explanatory features by target based on mean absolute SHAP value.

Target	Rank 1 feature (SHAP)	Rank 2 feature (SHAP)	Rank 3 feature (SHAP)
Like rate	Early interaction rate (0.118)	Initial exposure (0.091)	Semantic/category proxy (0.056)
Comment rate	Sentiment polarity (0.082)	Early comment rate (0.079)	Topic controversy (0.064)
Share rate	Hashtag diversity (0.074)	Content similarity (0.069)	Topic category (0.052)
Favorite rate	Informational density (0.088)	Creator history (0.076)	Subtitle length (0.047)
Completion rate	Video duration (0.102)	Retention-tempo proxy (0.086)	Duration-retention proxy (0.058)

This table emphasizes that explanatory importance is target-specific and helps relate computational salience to theoretically meaningful content and exposure mechanisms.

**Figure 10:**
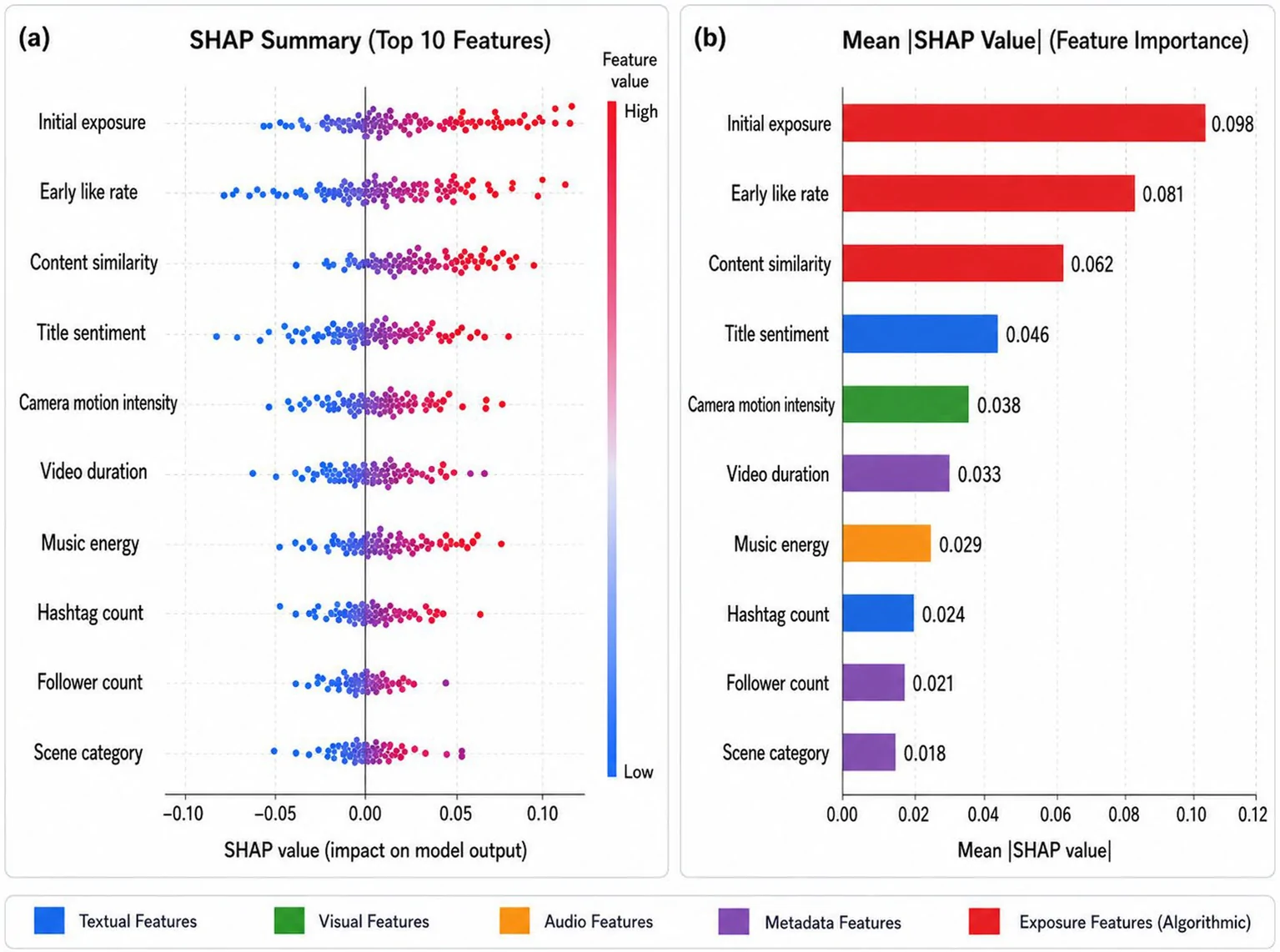
SHAP-based explanation of the engagement model. Panel **(a)** shows the distribution of local SHAP values for the top features, and Panel **(b)** reports the corresponding global mean absolute SHAP values. The figure reveals not only which variables matter most overall, but also how their contribution direction and variability differ across individual observations.

Figure 10 complements the tabular ranking by visualizing both the magnitude and the direction of feature contributions. Higher initial exposure and stronger early interaction signals shift model predictions upward, while the spread of SHAP points reveals substantial heterogeneity across videos. The same nominal exposure level has different implications depending on content category, creator size, and the co-occurrence of other modalities. This is important for communication research because it prevents an overly deterministic reading of platform effects. The algorithm matters, but it does not act in isolation; rather, it is associated with selective amplification of content that already carries certain advantageous multimodal signals.

The amplification hypothesis is evaluated through the interaction coefficient $\beta_{3}$ in Equation (21). Table 15 reports the interaction-model results. A positive and significant interaction term indicates that the association between content attractiveness and engagement becomes stronger under higher exposure intensity. This regression-based evidence is interpreted as a robustness check for the AAG analysis: AAG measures item-level amplification relative to the random-exposure baseline, whereas $\beta_{3}$ tests whether content effects are statistically moderated by observable exposure intensity.

**Table 15:** Interaction-model results for observable algorithmic amplification.

Predictor	Like rate	Comment rate	Share rate	Favorite rate	Completion rate
Content attractiveness $Q_{i}$	0.021^***	0.006^**	0.004^*	0.007^**	0.048^***
Exposure intensity $E_{i}$	0.037^***	0.011^***	0.009^***	0.013^***	0.032^***
$Q_{i}$ × $E_{i}$	0.018^***	0.005^**	0.004^**	0.006^**	0.021^***
Duration control	Yes	Yes	Yes	Yes	Yes
Category fixed effects	Yes	Yes	Yes	Yes	Yes
Time-window fixed effects	Yes	Yes	Yes	Yes	Yes
Adjusted *R*^2^	0.421	0.368	0.339	0.391	0.455

The table tests whether the association between content attractiveness and engagement becomes stronger as exposure intensity increases. ^*p* < 0.05, ^*p* < 0.01, ^*p* < 0.001. Values are reported estimates.

It is also noteworthy that the interaction coefficients are strongest for like rate and completion rate. This result indicates two partially distinct amplification pathways. The first is a rapid-reaction pathway, in which visible early appreciation increases the probability of additional low-cost engagement such as likes. The second is an attention-retention pathway, in which higher exposure is associated with more viewing opportunities while the internal structure of the video determines whether viewers remain long enough to complete it. Comment and share outcomes also exhibit positive interactions, but the smaller coefficients imply that these behaviors depend more heavily on higher-level social meaning, controversy, and perceived relevance. In other words, higher exposure is associated with amplified patterns across all targets, but it does not amplify them in exactly the same way.

**Figure 11:**
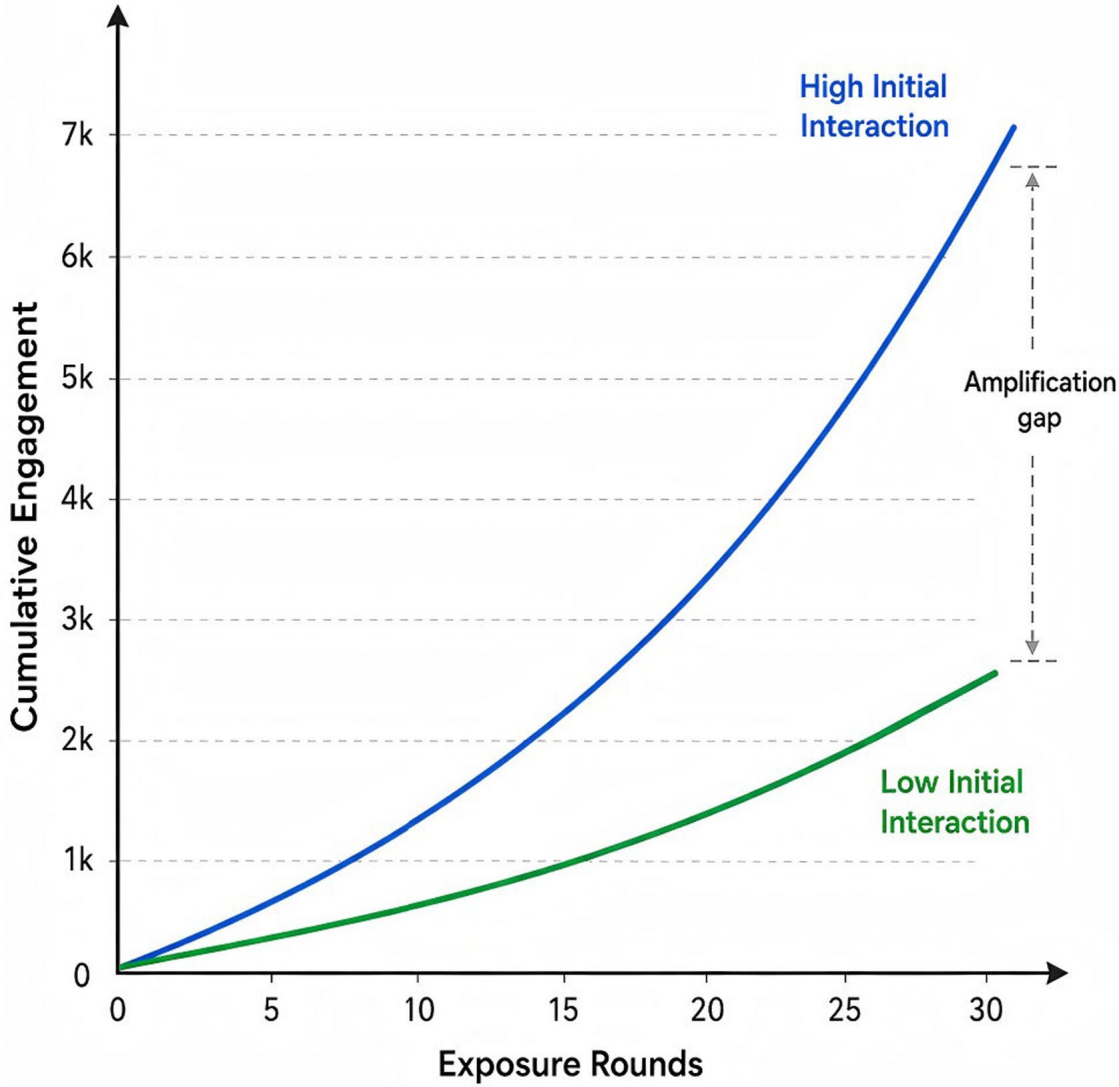
Empirical visualization of observable algorithmic amplification. The curves are aggregated from timestamped interaction logs by grouping videos into early-exposure/early-feedback strata and computing a normalized cumulative engagement index over exposure rounds. The widening gap indicates that small early differences in interaction are associated with substantially different engagement trajectories after repeated recommendation exposure; it should be interpreted as an observable pattern rather than proof of the proprietary ranking mechanism.

Figure 11 visualizes observable amplification patterns using aggregated trajectories from the timestamped interaction logs. Videos are grouped by early exposure and early feedback tertiles after controlling for category and duration. The cumulative engagement index is then computed for each exposure round by normalizing likes, comments, shares, favorites, and completion signals and averaging within each group. The resulting curves show that videos with comparable content-side scores diverge substantially when their early exposure and feedback conditions differ.

The substantive interpretation is as follows. First, exposure variables are not merely controls; they are part of the observable process by which content becomes visible. Second, engagement targets differ in their modality sensitivity. Like rate may respond to immediate affective and visual cues, comment rate may respond to sentiment or controversial topics, share rate may be related to topic relevance and social utility, favorite rate may reflect informational value, and completion rate may be affected by duration, semantic category, and retention-related viewing behavior. Third, explainable modeling helps translate prediction into human–media interaction theory. For instance, the stronger contribution of subtitles and information density to favorite rate than to share rate indicates that informational value encourages saving behavior more than diffusion behavior.

**Figure 12:**
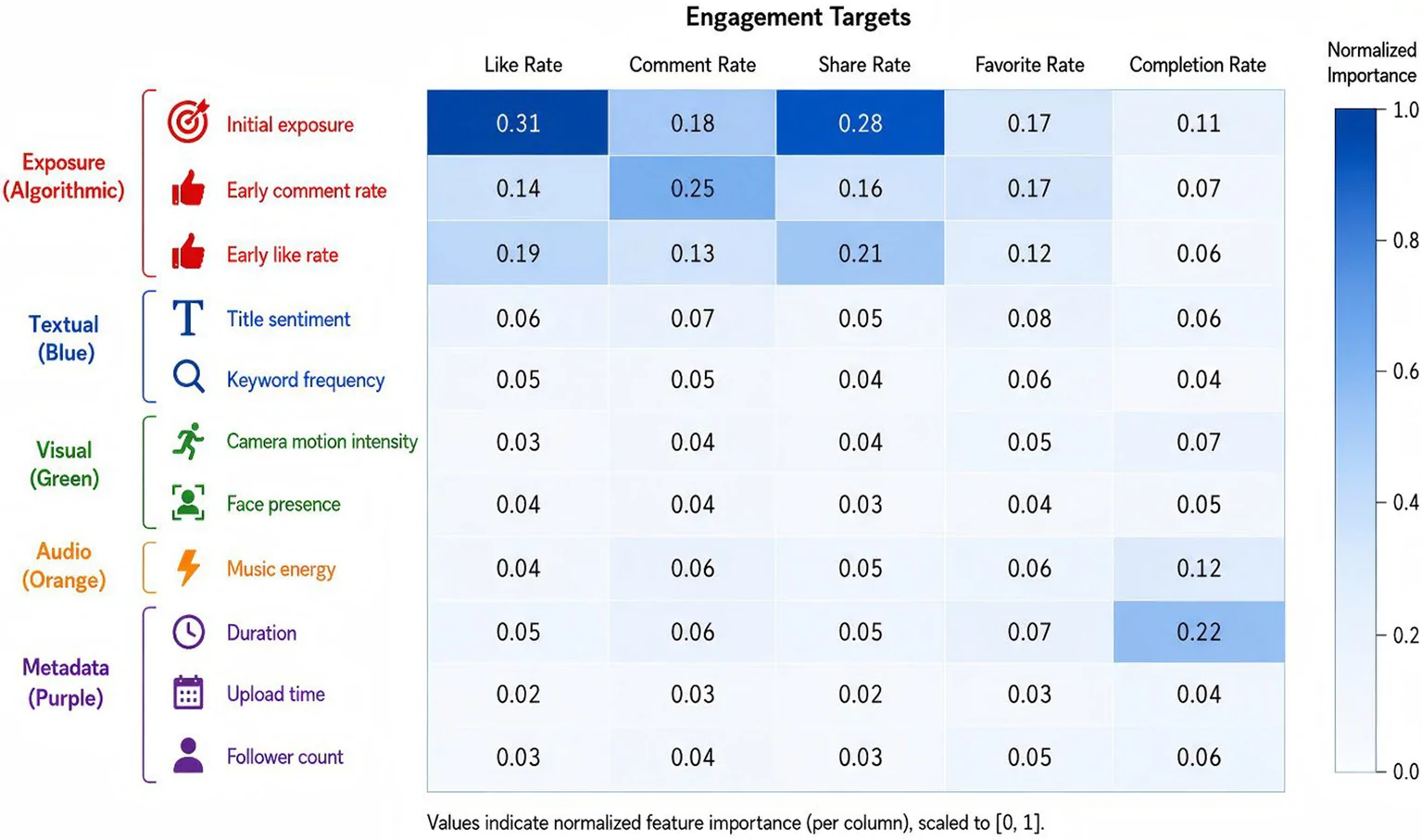
Cross-target feature-importance heatmap based on normalized mean absolute SHAP values. Each cell reports the contribution of a feature group to a target after column-wise normalization and rounding. Exposure-related variables dominate social-circulation behaviors, whereas duration and temporal/retention-behavioral proxy features contribute more strongly to completion, revealing both shared and behavior-specific drivers of engagement.

Figure 12 summarizes this cross-target heterogeneity in a compact way. The heatmap values are normalized mean absolute SHAP scores aggregated by feature group and target, with each column scaled to sum to one and rounded to two decimal places. The strongest cells concentrate in the exposure-related rows for like, comment, and share outcomes, whereas completion places relatively more weight on duration and temporal/retention-behavioral proxy features. This pattern is theoretically meaningful because it indicates that platform visibility primarily governs whether a video enters social circulation, while sustained attention depends more heavily on observable retention-related content structure.

### 4.3 Robustness checks and human–media interaction implications

Robustness checks are necessary because engagement data are noisy and platform-specific. Table 16 reports the observed evidence from the robustness tests used to examine the stability of the findings, including alternative engagement thresholds, category-specific models, duration controls, and exposure-only versus content-only comparisons.

**Table 16:** Robustness-check results.

Robustness test	Robustness specification	Observed evidence	Interpretation
High-engagement threshold sensitivity	Top 20%, top 25%, and top 30% thresholds	EGMF and LightGBM remained the two strongest classifiers under all three thresholds, and the AUC/F1 ordering changed only marginally.	The high-engagement conclusion is not driven by the selected percentile cut-off.
Duration control	Duration buckets and duration–feature interactions included	Completion-rate coefficients and SHAP rankings changed after duration controls, whereas like-rate and comment-rate rankings remained nearly unchanged.	Watch-related outcomes are duration-sensitive, while social reactions are more stable.
Category-specific modeling	Separate models trained for major first-level content categories	Exposure proxies were consistently important across categories, while textual and temporal/retention-behavioral proxy features showed stronger category-specific variation.	Engagement mechanisms are shared at the exposure level but heterogeneous at the content-modality level.
Content-only vs. exposure-only	Exposure-proxy-only, content-only, and full models compared	The full model achieved the highest performance across all five engagement targets; removing either content features or exposure proxies reduced predictive accuracy.	Content and exposure variables provide complementary explanatory power.
Temporal split	Early-period training and later-period testing	Temporal evaluation produced a moderate performance decline, but the leading features remained early interaction, initial exposure, duration, and semantic/category and temporal/retention-behavioral proxy descriptors.	The model captures patterns that generalize across adjacent time windows.
Outlier treatment	Top 1% view counts winsorized	The direction of the main coefficients and the top SHAP features remained consistent after winsorization.	The findings are not dominated by viral outliers.

The table reports observed evidence from alternative thresholds, duration controls, category-specific models, feature-block comparisons, temporal validation, and outlier treatment. These checks show that the main conclusions remain stable across different empirical specifications rather than depending on a single modeling choice.

Taken together, the robustness checks strengthen the credibility of the framework in three ways. First, stability across alternative thresholds indicates that the main conclusions are not an artifact of a particular operational definition of “high engagement.” Second, temporal and category-specific splits test whether the models merely memorize short-term platform regularities or capture more general multimodal patterns. Third, the comparison between content-only and exposure-only models is especially important for theoretical interpretation. If exposure-only models performed nearly as well as the full model, one might conclude that visibility dominates content. If content-only models were similarly competitive, then multimodal content characteristics would remain the primary explanation. The observed pattern is complementarity: neither block alone is sufficient, while their combination yields the most complete account of user engagement.

The human–media interaction implications are broader than model performance. The findings can be interpreted through the transformation of the content production–distribution–feedback chain. In a traditional media environment, distribution is relatively separated from feedback: audiences respond after content is distributed. In short-video platforms, feedback is immediately folded back into distribution. A video that receives early likes, comments, or completion may be associated with broader subsequent recommendation exposure, which can then coincide with more feedback. Therefore, creators are not only competing for audience attention; they are competing for algorithmic recognition. This changes the logic of content production. Titles, subtitles, semantic/category cues, retention rhythm, and topic tags are not simply expressive devices; they are signals that may interact with platform ranking and exposure allocation.

The proposed framework also helps explain why different engagement behaviors should not be collapsed into one popularity score. A video may be liked but not shared, commented on but not completed, or completed but not favorited. Each behavior reflects a different communication function. Likes indicate low-cost approval; comments indicate conversational participation; shares indicate social diffusion; favorites indicate perceived future value; completion indicates sustained attention. A multi-target framework is therefore more suitable than a single aggregate engagement index.

From a platform-governance perspective, the framework can also support algorithmic auditing. If exposure proxies dominate engagement prediction, platform visibility may have a stronger role than content quality alone. If certain categories or creator types receive systematically different exposure after controlling for content and early feedback, further fairness or diversity analysis may be required. Although the present study does not directly access proprietary ranking algorithms, it provides a measurable way to examine how observable exposure patterns are associated with engagement outcomes.

## 5 Conclusion

This paper presents an explainable exposure-aware framework for short-video engagement prediction and observable algorithmic amplification measurement. The framework models short-video feeds as human–media interaction systems in which content representations, metadata, exposure conditions, and early feedback jointly shape later user behavior. By combining KuaiRand-Pure, KuaiRec, and MicroLens, the study connects exposure-aware recommendation logs with external raw-multimodal validation.

The main contribution is methodological. The proposed AAG index uses random-exposure logs to estimate a content-side engagement baseline and compares it with engagement observed under standard recommendation. This design provides a reproducible proxy for observable recommendation-associated amplification without requiring access to proprietary ranking parameters. The EGMF component further tests whether visibility/context representations can condition the contribution of content-side feature groups. The results show that exposure proxies and early feedback are consistently important, but textual, semantic/category proxy, temporal/retention-behavioral proxy, and raw multimodal features remain necessary for target-specific engagement explanation.

The findings also support a multi-target view of short-video interaction. Like, comment, share, favorite, and completion behaviors are not interchangeable signals. Likes often reflect low-cost approval, comments reflect conversational participation, shares reflect social diffusion, favorites reflect perceived future utility, and completion reflects sustained attention. A single popularity score would therefore hide important differences in how users interact with algorithmically mediated video content.

The study has limitations. KuaiRand-Pure provides random-exposure records, but the internal Kuaishou ranking function is not observed. The exposure variables are therefore transparent mechanism proxies rather than direct platform parameters, and the AAG should be interpreted as a proxy measure of observable amplification rather than a definitive causal estimate. Unobserved user preferences, temporal context, creator-level reputation, seasonal events, and content life cycles may also influence both exposure and engagement. In addition, KuaiRand-Pure and KuaiRec are both derived from Kuaishou, while MicroLens offers external validation under a different benchmark structure. Future research can extend the framework through platform-side randomized experiments, longitudinal creator-level tracking, causal-inference designs, fairness-aware exposure auditing, and cross-platform comparison.

Overall, the paper shows that short-video engagement prediction can be treated not only as a recommender-system task but also as an explainable human–media interaction problem. The proposed framework provides a reproducible way to examine how algorithmic visibility, multimodal content, and user feedback are jointly associated with engagement on short-video platforms.

## Data Availability

The original contributions presented in the study are included in the article/Supplementary material, further inquiries can be directed to the corresponding author.
