# Supplementary material for: Exposure-aware multimodal engagement prediction for short-video platforms: an explainable human–media interaction framework
Source: Front Artif Intell. 2026 Aug 25;9:1896199. doi: 10.3389/frai.2026.1896199 (PMC13550198; doi:10.3389/frai.2026.1896199)
Supplement: Supplementary file 1 [file Data_Sheet_1.docx]

Figure Prompts

The photographs, video thumbnails, user-interface mock-ups, and human figures visible in Figures 1–6 are illustrative AI-generated elements rather than photographs of study participants, screenshots collected from individual users, or images reproduced from third-party publications

The image-generation process was iterative. As the original instructions were provided through several rounds of layout and visual refinement, the consolidated prompts below reproduce the substantive instructions given to ChatGPT:

1. Figure 1

“Create a two-panel academic infographic comparing a traditional linear communication chain with an algorithmic short-video environment. In the left panel, show content production, platform distribution, audience reception, and user feedback as a one-way sequence. In the right panel, show multimodal content, initial exposure, user engagement, feedback signals, algorithmic re-ranking, and further exposure as a recursive feedback loop. Use generic illustrative content and interface mock-ups on a clean white background.”

2. Figure 2

“Create a three-part academic infographic explaining the algorithmic amplification gap. The first section should compare a uniform random-exposure log with a standard recommendation log. The second section should show shared content descriptors, a natural-response estimator, observed engagement under recommendation, and the equation AAG = observed engagement minus expected natural engagement. The third section should show low, neutral, and high amplification groups and applications to amplification diagnosis, creator fairness, and robustness analysis. Use representative generic video thumbnails and simplified statistical charts.”

3. Figure 3

“Create an academic framework diagram for exposure-aware and explainable short-video engagement prediction. Organize the diagram into a content layer, an exposure layer, and a feedback layer. Include textual, visual, audio, and metadata features; impression intensity, early interaction signals, and content-user matching; and engagement outcomes such as like, comment, share, favorite, and completion rates. Connect these components to an explainable machine-learning module and interpretation outputs such as SHAP, feature importance, and partial-dependence plots.”

4. Figure 4

“Create a six-stage methodological workflow for short-video engagement research. Show public short-video sampling, data cleaning and screening, multimodal feature extraction, engagement-target construction, model training, and performance evaluation. Include representative interface mock-ups, data-cleaning indicators, textual, visual, audio, metadata, and exposure features, engagement-rate formulas, machine-learning models, and evaluation metrics. Present the stages from left to right using a clean academic infographic style.”

5. Figure 5

“Create a structured multimodal feature-system diagram for short-video engagement modeling. Organize the feature space into five groups: textual features, visual features, audio features, metadata features, and algorithmic-exposure proxy features. Show representative feature examples for each group, followed by feature vectorization, fusion and concatenation, normalization, machine-learning models, and the five engagement targets: like, comment, share, favorite, and completion rates.”

6. Figure 6

“Create an academic architecture diagram for an Exposure-Gated Multimodal Fusion model. Show six stages: input features, modality projection, exposure gate, gated fusion, prediction backbone, and interpretation and outputs. Include textual, visual-semantic, temporal or behavioral, metadata, and exposure features. Show exposure-derived gate weights regulating the contribution of the other modality representations before multi-target engagement prediction. Add interpretation outputs including SHAP, feature importance, and calibration.”
